# Unveiling the neuroprotective potential of *Ipomoea carnea* ethanol extract via the modulation of tau and *β*-secretase pathways in AlCl_3_-induced memory impairment in rats in relation to its phytochemical profiling

**DOI:** 10.1007/s10787-025-01687-0

**Published:** 2025-03-12

**Authors:** Walaa A. El-Kashak, Ahmed F. Essa, Mohamed F. Abdelhameed, Yasmine H. Ahmed, Asmaa S. Abd Elkarim, Mai M. Elghonemy, Bassant M. M. Ibrahim, Ahmed H. Gaara, Tahia K. Mohamed, Abdelsamed I. Elshamy

**Affiliations:** 1 Chemistry of Natural Compounds Department, National Research Centre, 33 El Bohouth St, Dokki, Giza, 12622 Egypt; 2 Pharmacology Department, National Research Centre, Dokki, 12622 Giza Egypt; 3 Department of Cytology and Histology, Faculty of Veterinary Medicine, Cairo University, Giza, 12211 Egypt; 4 Chemistry of Tanning Materials and Leather Technology Department, National Research Centre, 33 El Bohouth St., Dokki, 12622 Giza Egypt

**Keywords:** Alzheimer’s disease, *Ipomoea carnea*, AlCl_3_, Tau, *β*-secretase pathway

## Abstract

Alzheimer’s disease (AD) is a widespread condition that affects adults and the community considerably. The causes are yet unknown, except from advanced age and genetic predisposition. Natural products provided advantageous advantages for managing AD due to their efficacy, safety, and accessibility. The memory boosting effects of chemically characterized *Ipomoea carnea* ethanol extract (IPC-EtOH) on behavioral, biochemical, histological, and molecular levels against cognitive impairment induced by AlCl_3_ exposure in rats were assessed using donepezil as a reference drug. Behavioral tests (spontaneous alternation T-maze and open field test) and assays for GSK3β, CREB, FOXO1a, β-secretase, tau, oxidative stress biomarkers, histopathology, and immunohistochemistry for cyclooxygenase 2 (COX-2) were conducted. The chemical profiling of IPC-EtOH using UPLC-ESI–qTOF-MS coupled with molecular networking revealed the identification of 83 bioactive metabolites, including pyrrolizidine alkaloids and cinnamic acid derivatives which previously undescribed from this species. AlCl_3_ injection significantly elevated tau, *β*-secretase, GSSG, GSK-3*β*, and FOXO3a levels and down regulated CAT, SOD, and CREB, with strong COX-2 immunoexpression in the cortex and hippocampus compared to controls. Oral co-administration of donepezil or IPC-EtOH to AlCl_3_-treated rats restored near-normal function in these brain regions, significantly attenuating spatial learning, memory, and locomotor impairments. These results suggest that IPC-EtOH could be a promising therapy for mitigating aluminum-induced neurotoxicity, though further studies are needed to elucidate its precise mechanisms of action. These outcomes emphasize *I. carnea* ethanol extract’s potential as an appealing therapy for AD by demonstrating its neuroprotective and memory-enhancing properties in rats having AlCl_3_-induced memory impairment.

**Supplementary Information:**

The online version contains supplementary material available at 10.1007/s10787-025-01687-0.

## Introduction

Among neurodegenerative disorders, Alzheimer’s disorder (AD) is the most widespread form of dementia that causes diminished memory along with attention problems. Over forty-four million individuals worldwide were diagnosed with AD in 2015; however, by 2050, the number of cases is predicted to quadruple to 115,000,000 (Zhao et al. 2020). Neurofibrillary tangles (NFTs), senile plaques, and brain atrophy, primarily in the areas of the hippocampus, the parietal and temporal lobes are characteristic features of AD. The build-up of beta-amyloid (Aβ) has been demonstrated to cause oxidative damage and neurological damage, both of which are linked to the advancement of AD. Although the exact causes of AD are undetermined, a number of investigations have suggested that aluminum may be a causal agent (Zhao et al. 2020; Ibrahim et al. 2024).

Aluminum is a common metal in environments and can be readily absorbed by the human organism through antacids, instruments, water, and cosmetics. Aluminum is a hazardous heavy metal that builds up in the body and can have harmful effects on several tissues, including the liver, spleen, and brain (Willhite et al. 2014; Abbas et al. 2022). Therefore, the neuroprotective benefits of various phytochemicals and chemical compounds against AD can be studied using rats that have been treated with aluminum chloride (AlCl_3_) (Hamdan et al. 2022).

Despite the fact that the majority of developed nations have access to basic AD treatment options, the therapeutic index is still insufficient to reverse the condition. In underdeveloped nations, many cannot even buy generic medications, despite the fact that they are ineffective. Pharmaceutical treatments are excessively costly and difficult to obtain, thus, chemically manufactured medications may be risky and have adverse effects that are undesirable (Shunan et al. 2021). A major curiosity in beneficial management studies with an appropriate natural product for managing AD in terms of medication effectiveness, availability, and safety was sparked by the development of the prevalence and the correlation between disabilities and the frightening health quality with a higher death rate in both developing and developed countries. According to reviews published recently, patients on a therapeutic diet are less likely to have AD (Ismail and Mirza 2015).

Around 600 botanical species pertaining to the *Ipomoea* genus (family: Convolvulaceae) are planted as decorative, weed, and medicinal herbs around the globe (Meira et al. 2012). There is evidence that some *Ipomoea* plants offer dietary benefits; *I. batatas*, for example, is commonly known as the sweat potato throughout globe (Abd-ElGawad et al. 2022). Numerous metabolites, including as terpenes, flavonoids, coumarins, lignans, and alkaloids, have been characterized throughout the documented studies of these plants (Kourouma et al. 2020; Abd-ElGawad et al. 2022).

A common plant in many nations throughout the globe utilized in folk medicine is *I. carnea* Jacq. As well as being used as a laxative and to provoke menstruation, various parts of *I. carnea* were also documented to be utilized in traditional remedies to treat a variety of illnesses, including gout, rheumatism, immune deficiency, dysentery, and skin and venereal problems (Wadnerwar and Deogade 2021). Furthermore, a variety of pharmaceutical functions of the *I. carnea* extracts have been deduced and documented, which include antibacterial, antifungal, cytotoxic, antioxidant (Ambiga and Jeyaraj 2015; Abd-ElGawad et al. 2022), hypoglycemic, immune-regulating (Hueza and Górniak 2011), wound healing (Shukla et al. 2018), anticonvulsant, anxiolytic, anti-inflammation, a relaxant, and anti-liver toxicity (Wadnerwar and Deogade 2021). The previous chemical characterization of the extracts and oils derived from *I. carnea* exhibited that it contains alkaloids, polyphenolic components, including phenolic acids, flavonoids, and tannins, in addition to the terpenoids, sterols, and saponins (Khan et al. 2019; Abd-ElGawad et al. 2022).

Consequently, the current investigation was designed to perform the following tasks: (i) assessment the protective impact of *I. carnea* ethanolic extract (IPC-EtOH) toward AlCl_3_-induced memory deficits in the rat model; (ii) profiling the metabolites of IPC-EtOH using multiplex of ultra-performance liquid chromatography–electrospray ionization–tandem mass spectrometry (UPLC-ESI–qTOF-MS) and molecular networking to comprehensively characterize a wide range of metabolites; and (iii) providing the mechanisms of action underlying the neuroprotective abilities of IPC-EtOH throughout the inflammation detection in the cerebral cortex and hippocampus based upon the behavioral, biochemical, pathological, and immunohistochemical assessments.

## Materials and methods

### Plant collection, authentication and preparation

The aerial parts of *I. carnea* were gathered in early morning (4–6 AM) on 18 April 2022 from the canals of the cultivation lands near Mansoura governorate (31° 04029.400 N 31° 25,005.300 E), Egypt. The plant specimen was recognized and verified via Prof. Ahmed M. Abd-ElGawad, taxonomy professor in the Faculty of Science, Mansoura University. The voucher specimen was also generated and delivered with voucher code number IPC-66-XfTTR-2022 to the Mansoura University Herbarium.

### Extraction processing

After 10 days of air drying at room temperature in an open, shaded area, the plant parts were ground up and stored in an absorbent paper bag to be prepared for extraction. The air-dried plant material (800 g) was macerated in a mixture of ethanol and distilled water in a ratio of 7:3 (4 L) for one week and then filtered. The filtrate was re-extracted by the same process two times. The extracts in liquor form were collected and dried under vacuum at temperature of 45.0 °C, affording black gum (26.3 g). The extract was kept in the refrigerator at − 4 C until the starting of the pharmacological experiments.

### UPLC-ESI–qTOF-MS chemical profiling of IPC-EtOH

One gram of powdery *I. carnea* was extracted for one hour employing an ultrasonic bath (Branson Ultrasonic Corporation, Danbury, CT, USA) with a 70% hydro-ethanol mixture. After filtration, the resultant solution had been centrifuged for 15 min. The extract liquid's clear supernatant was then separated and subjected to UPLC-ESI-QTOF-MS analysis by the same procedure described before (Ibrahim et al. 2024). To summarize, 10 mg of the dry and coarsely crushed plant material were extracted employing 20 min of ultrasound with regular shaking. An internal norm of 10 g/mL^−1^ umbelliferone was also utilized. Two milliliters of 100% MeOH as the solvent were utilized. The centrifuge for 10 min at 12,000 × g cleared the trash. The 22-μm-filtered extract was then subjected to extraction in solid phase using a C18 cartridge. The plant extract was subsequently added in the amount of 2 μL to an HSS T3 column (100 × 1.0 mm, particle size 1.8 μm; Waters) that was mounted on an ACQUITY UPLC system (Waters, Milford, MA, USA) equipped with a 6540 Ultra-High-Definition (UHD) Accurate-Mass qTOF-LC–MS (Agilent, Palo Alto, CA, USA) that could be operated in any direction and was connected to an ESI interface. The metabolites were found by developing a possible formula with a mass accuracy limit of 10 ppm that took into consideration tandem MS2 data, RT, literature research, and the Phytochemical Dictionary of Natural Products Database (Kabbash et al. 2023).

### Molecular networking

The raw data files generated by UPLC-ESI-QTOF-MS/MS in both modes were converted to mzML format using the MS Convert tool from ProteoWizard (version 3.0.21050, available at https://proteowizard.org). The processed files were then transferred to the MassIVE repository via WinSCP (https://massive.ucsd.edu). Molecular networking (MN) of the LC-QTOF-MS/MS data was performed using the Global Natural Product Social (GNPS) MN platform. The analysis employed a precursor ion mass tolerance of 0.2 Da and a fragment ion tolerance of 0.05 Da, adhering to the default GNPS networking parameters. A minimum of six MS fragment matches between two consensus MS/MS spectra was required, with a cosine similarity score of at least 0.7 to establish connections between nodes. The Graph ML file of the resulting network was subsequently downloaded and visualized in a force-directed layout utilizing the Cytoscape 3.7.1 software (Essa et al. 2023).

### Drugs and chemicals

Donepezil (CAS No.: 120011–70-3) and aluminum chloride (AlCl_3_; CAS No.: 7446–70-0) were acquired from Sigma-Aldrich Co. Inc. (St. Louis, MO, USA). Saline was used to dissolve them. Additional chemicals of the highest analytical purity were employed. β-Secretase Fluorometric Assay Kit (Cat #K360-100), Catalase (CAT, Cat #K773-100), and Superoxide Dismutase (SOD, Cat #K335-100) colorimetric kits were purchased from Biovision, CA, USA. Oxidized Glutathione (GSSG, Cat # EK721431) ELISA Kit was obtained from AFG Bioscience, Northbrook, USA. Rat Tau ELISA Kit (Cat # NBP2–81,164, Novus Biologicals, LLC, USA), Rat FOXO1A ELISA Kit (Cat # A303779, antibodies-online Limerick PA USA). The remaining ELISA kit, Glycogen synthase kinase-3 beta (GSK3β, Cat # ER0060) and Cyclic AMP Response Element Binding Protein (CREB, Cat # ER0865), were obtained from FineTest^®^, Boulder, USA. All procedures were performed according to the manufacturer’s guidelines.

### Animals and ethical statements

Female Wistar rats aged 12 weeks and weighed between 165 and 185 g were purchased from the National Research Center (NRC)’s animal house in Cairo, Egypt. Before the trial began, the animals were acclimated for 1 week at NRC’s animal facility. The rats were kept in a setting that was tracked for temperature (23 ± 2 °C), humidity (60 ± 10%), and light/dark cycling (12/12 h). They were additionally provided with both water and food on demand. The National Research Center (NRC) Ethics Committee evaluated and approved the investigative methods (Ethical permission no: 13060134). Furthermore, the procedure adhered to the National Institutes of Health’s (2011) Guide for the Maintenance and Utilization of Laboratory Animals. Every effort was made to lessen the pain that the animals endured during the research investigations.

### Experimental design

A total of 35 female rats were randomly assigned to five groups, with 7 rats in each group. Group I was given an ordinary diet and given daily 0.5-ml 0.9%-NaCl solution orally and was considered the normal control group. Group II (positive control group) involved injecting 0.5 ml of AlCl_3_ at a dosage of 172 mg/kg intraperitoneally into rats for a duration of 4 weeks for the induction of an animal model mimicking AD (Zhao et al. 2020; Shunan et al. 2021; Hamdan et al. 2022). The rats in group III (reference drug group) received everyday oral doses of 5 mg/kg of donepezil, as a reference drug (Ademosun et al. 2022; Ibrahim et al. 2024), one hour before AlCl_3_ injection for 4 weeks. Rats in groups IV and V received daily oral doses of 100 and 200 mg/kg IPC-EtOH, respectively, one hour before receiving an AlCl_3_ injection as group II for a period of 4 weeks. Behavioral assessments were carried out to assess the degree of impairment in memory and spatial recognition at the completion of the 4-week investigation.

### Behavioral evaluation

After 24 h of the last therapeutic dose, behavioral stress assessments were conducted, including spontaneous alternation T-maze and open field tests.

### Spontaneous alternation T-Maze

All of the rats’ continuous spontaneous alteration behavior was assessed utilizing the T-maze apparatus, which measures the rats’ spatial working memory, short-term memory, and capacity for exploration. Normative rats will be quicker entering the new arm of the T-maze than returning to the one they had already explored. The equation below illustrates the percentage of spontaneous alternation (Ijomone et al. 2015; Mititelu-Tartau et al. 2024).

$$\text{Spontaneous alternation\%}=\frac{\text{Number of alternations}}{\text{Number of choices}-2} X 100$$

### Open field test

The open experiment is employed to assess the rats’ psychological state. For 5 min, each rat was observed, and the number of squares it crossed was recorded (Fragkiadaki et al. 2024).

### Biochemical assays

After the experiments (4 weeks) were over, the rats were mercifully and swiftly murdered by cervical dislocation while anesthetized with an intraperitoneal injection of 40–50 mg/kg sodium pentobarbital. The rats were eventually disposed of in accordance with the instructions issued by the Safety and Health Committee of the National Research Center (NRC).

After being quickly dissected, each animal’s entire brain was properly cleaned with isotonic saline, dried, and weighed. Each brain was then agitatedly split into two halves. Each brain’s initial part was homogenized right away, yielding a 10% (w/v) homogenate in an ice-cold medium with 300 mmol/l sucrose and 50 mmol/l Tris–HCl (pH 7.4). The homogenate was centrifuged for 10 min at 41 °C at 3000 rpm. For biochemical examination (GSSG, CAT, SOD, GSK3β, CREB, FOXO1A, β-secretase, and tau), the 10% supernatant was isolated. Utilizing Elisa Kits, all biochemical parameters were detected.

### Histopathological examination

#### Light microscopic investigation

Brain tissue was obtained and fixed for 48 h in 10% neutral buffered formalin. The sections were then dehydrated in ascending concentrations of ethyl alcohol, cleared in xylene, and embedded in paraffin wax. Four-µm thick sections were produced, deparaffinized, and stained with hematoxylin and eosin (H&E) for light microscopic examination (Bancroft and Gamble 2008). Other tissue sections were used for immunohistochemical examination.

#### Immunohistochemical examination of Cyclooxygenase 2 protein (COX-2)

COX-2 was used in this study for the detection of inflammation in the cerebral cortex and hippocampus. The method used in this study was outlined by Côté 1993** (**Côté 1993**).** Anti-COX-2 immunohistochemical sections were used to measure the mean area on different slides (n = 5 fields/group) using image analysis software (LEICA microsystems (LAS version 3.8.0 (build: 878), Leica Ltd) image analyzer computer system) at the Cytology and Histology Department, Faculty of Veterinary Medicine, Cairo University.

### Statistical analysis

The results are expressed as mean ± SD. Data were analyzed using one-way analysis of variance (ANOVA) to determine the significance of the mean between the groups, followed by Tukey’s post-hoc test. Statistical significance was set at *P* < 0.0001.

## Results

### Phytochemical profiling of IPC-EtOH

**Fig. 1:**
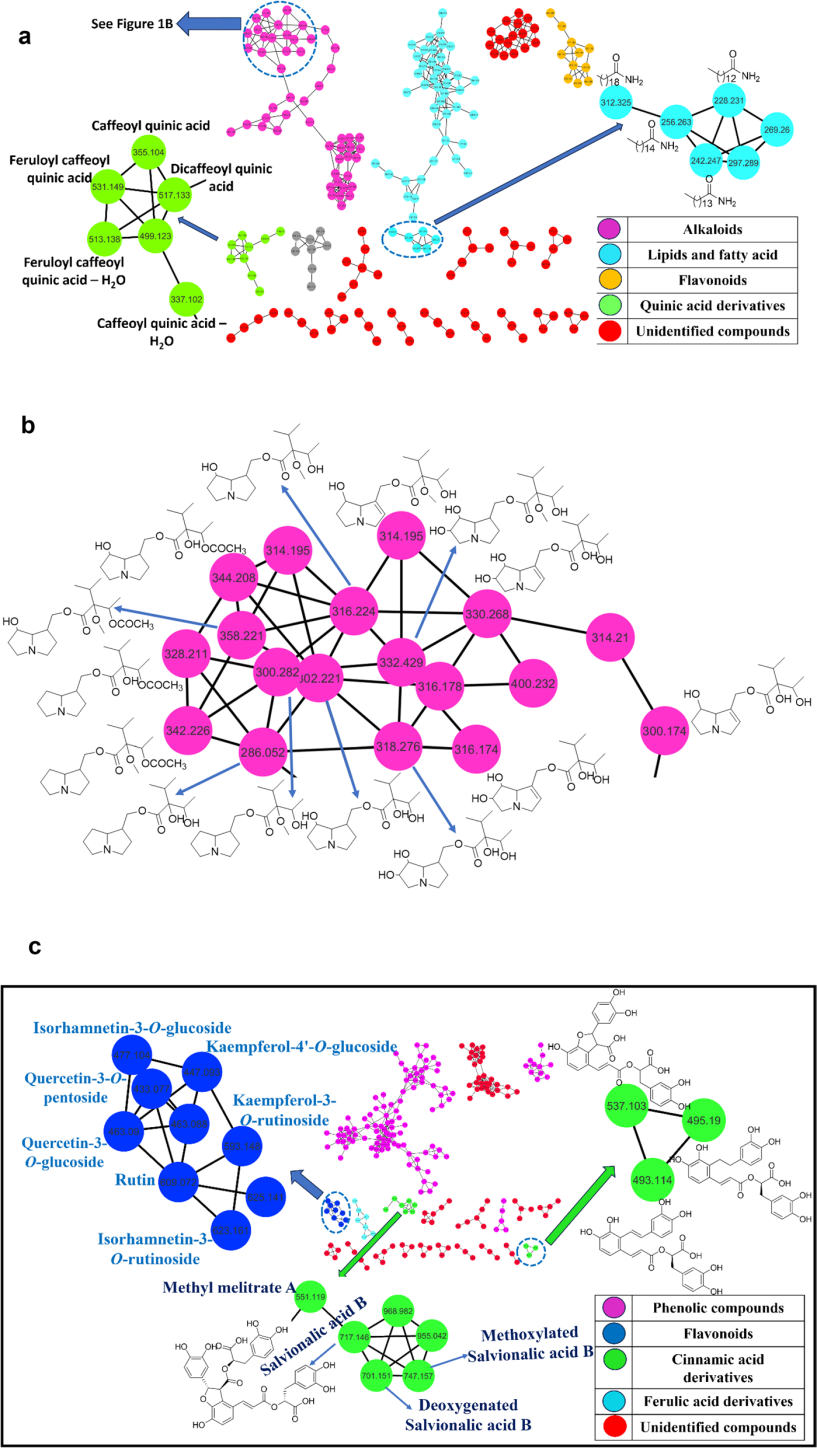
**A** UPLC-ESI–MS-based molecular networking in positive ionization mode of the extract of *Ipomoea carnea*. **B** Molecular network of the molecular family of alkaloids extracted from the MN of the extract of *Ipomoea carnea*. **C** UPLC-ESI–MS based molecular networking in negative ionization mode of the extract of *Ipomoea carnea*

The chemical profile of *I. carnea* ethanol extract (IPC-EtOH) was annotated using multiplex of LC–ESI–MS-MS technique and molecular network (Fig. 1). Both positive and negative ionization modes (Fig. 1S) were used. The tentative identification of alkaloids, phospholipids, fatty acid derivatives, phenolic compounds, coumarins, and others was achieved by comparing Rt, molecular weight, and the MS–MS fragmentation pattern with previously known constituents (Table 1).

**Table 1:** Tentative identification of bioactive secondary metabolites in *Ipomoea carnea* ethanol extract using UPLC-ESI-QTOF-MS

No	Rt min	m/z	MS^n^ product ions	El. composition	Error ppm	Identification
*Pyrolizidine alkaloids*						
1	4.27	300.1796	156, 138, 120, 94	C_15_H_26_NO_5_^+^	3.16	7-Hydroxy-9-(2ʹ,3ʹ-dihydroxy-2ʹ-isopropylbutanoyl)1-hydroxymethyl dehydropyrrolizidine
2	4.66	316.1744	298, 172, 138	C_15_H_26_NO_6_^+^	3.33	6,7-Dihydroxy 9-(2ʹ,3ʹ-dihydroxy-2ʹ-isopropylbutanoyl)1-hydroxymethyl dehydropyrrolizidine
3	4.75	318.1900	300, 274, 174, 156, 138	C_15_H_28_NO_6_^+^	3.50	6,7-Dihydroxy-9-(2ʹ,3ʹ-dihydroxy-2ʹ-isopropylbutanoyl)1-hydroxymethyl pyrrolizidine
4	4.99	286.2004	268, 242, 142, 124	C_15_H_28_NO_4_^+^	3.06	9-(2ʹ,3ʹ-Dihydroxy-2ʹ-isopropylbutanoyl)1-hydroxymethylpyrrolizidine
5	4.94	284.1846	240, 140, 122	C_15_H_26_NO_4_^+^	3.43	9-(2ʹ,3ʹ-Dihydroxy-2ʹ-isopropylbutanoyl)1-hydroxymethyl dehydropyrrolizidine
6	4.95	302.1951	284, 158, 140	C_15_H_28_NO_5_^+^	3.44	7-Hydroxy-9-(2ʹ,3ʹ-dihydroxy-2ʹ-isopropylbutanoyl)1-hydroxymethyl pyrrolizidine
7	5.40	314.1951	270, 156, 138, 120, 94	C_16_H_28_NO_5_^+^	3.50	7-Hydroxy-9-(3ʹ-hydroxy-2ʹ-isopropyl-2ʹ-methoxybutanoyl)1-hydroxymethyl dehydropyrrolizidine
8	5.74	332.2056	314, 288, 174, 156, 138	C_16_H_30_NO_6_^+^	3.59	6,7-Dihydroxy-9-(3ʹ-hydroxy-2ʹ-isopropyl-2ʹ-methoxybutanoyl)1-hydroxymethyl pyrrolizidine
9	5.76	330.1899	286, 172, 138	C_16_H_28_NO_6_^+^	3.65	6,7-Dihydroxy-9-(3ʹ-hydroxy-2ʹ-isopropyl-2ʹ-methoxybutanoyl)1-hydroxymethyl dehydropyrrolizidine
10	5.89	360.2006	300, 282, 174, 156, 138	C_17_H_30_NO_7_^+^	5.99	6,7-Dihydroxy-9-(3ʹ-acetoxy-2ʹ-hydroxy-2ʹ-isopropylbutanoyl)1-hydroxymethyl pyrrolizidine
11	5.92	300.2159	282, 256, 142, 124	C_16_H_30_NO_4_^+^	3.51	9-(3ʹ-Hydroxy-2ʹ-isopropyl-2ʹ-methoxybutanoyl)1-hydroxymethyl pyrrolizidine
12	6.06	298.2004	140, 122	C_16_H_28_NO_4_^+^	3.53	9-(3ʹ-Hydroxy-2ʹ-isopropyl-2ʹ-methoxybutanoyl)1-hydroxymethyl dehydropyrrolizidine
13	6.06	316.2110	298, 158, 140	C_16_H_30_NO_5_^+^	3.07	7-Hydroxy-9-(3ʹ-hydroxy-2ʹ-isopropyl-2ʹ-methoxybutanoyl)1-hydroxymethyl pyrrolizidine
14	6.11	328.2108	268, 250, 142, 124	C_17_H_30_NO_5_^+^	3.35	9-(3ʹ-Acetoxy-2ʹ-hydroxy-2ʹ-Isopropylbutanoyl)1-hydroxymethyl pyrrolizidine
15	6.25	344.2058	284, 158, 140	C_17_H_30_NO_6_^+^	2.86	7-Hydroxy-9-(3ʹ-acetoxy-2ʹ-hydroxy-2ʹ-isopropylbutanoyl)1-hydroxymethyl pyrrolizidine
16	6.32	342.1901	282, 156, 138, 120, 94	C_17_H_28_NO_6_^+^	2.91	7-Hydroxy-9-(3ʹ-acetoxy-2ʹ-hydroxy-2ʹ-isopropylbutanoyl)1-hydroxymethyl dehydropyrrolizidine
17	7.20	342.2266	282,264, 142, 124	C_18_H_32_NO_5_^+^	2.75	9-(3ʹ-Acetoxy-2ʹ-isopropyl-2ʹ-methoxybutanoyl)1-hydroxymethyl pyrrolizidine
18	7.36	358.2214	298, 158, 140	C_18_H_32_NO_6_^+^	2.91	7-Hydroxy-9-(3ʹ-acetoxy-2ʹ-isopropyl-2ʹ-methoxybutanoyl)1-hydroxymethyl pyrrolizidine
19	7.81	374.2163	314, 296, 174, 156	C_18_H_32_NO_7_^+^	2.64	6,7-Dihydroxy-9-(3ʹ-acetoxy-2ʹ-isopropyl-2ʹ-methoxybutanoyl)1-hydroxymethyl pyrrolizidine
*Other nitrogenous compounds*						
20	1.67	188.0558	128, 102	C_7_ H_10_NO_5_^−^	2.3	N-acetyl glutamic acid
21	1.86	243.0617	200, 110	C_9_H_11_N_2_O_6_^−^	2.17	Arabinosyl uracil
22	1.88	290.0883	200, 128	C_11_ H_16_NO_8_^−^	4.1	N-fructosyl pyroglutamate
23	2.57	314.0901	182, 136, 97	C_11_H_16_N_5_O_4_S^+^	5.10	5ʹ-Deoxy-5ʹ-(methylsulfinyl) adenosine
24	2.61	268.1044	136	C_10_H_14_N_5_O_4_^+^	1.27	Adenosine
25	3.80	384.1138	252, 162, 136	C_14_H_18_N_5_O_8_^+^	3.09	Succinoadenosine
26	4.21	203.0817	116, 142	C_11_H_11_N_2_O_2_^−^	0.72	Tryptophan
27	4.72	413.1402	281, 162, 136	C_15_H_21_N_6_O_8_^+^	3.24	N6-Threonyl carbamoyl adenosine
28	6.35	206.0822	164, 147	C_11_H_12_NO_3_^−^	4.76	N-acetylphenylalanine
29	9.05	314.1378	177, 145, 121	C_18_H_20_NO_4_^+^	2.75	Feruloyltyramine
*Phospholipids and fatty acids*						
*Phosphatidylglycerol*						
30	14.13	483.27267	255, 153	C_22_ H_44_O_9_P^−^	1.9	PG (16:0/0:0)
31	17.00	745.5028	509, 255, 279	C_40_ H_74_O_10_P^−^	1.7	PG (16:0/18:2)
32	17.07	721.5034	255, 153	C_38_ H_74_O_10_P^−^	2.7	PG (16:0/16:0)
*Phosphatidylcholine*						
33	14.22	496.3386	184	C_24_H_51_NO_7_P^+^	2.31	PC (16:0/0:0)
34	16.98	758.5673	184	C_42_H_81_NO_8_P^+^	0.75	PC (16:0/18:2)
35	12.28	571.2888	315, 255, 241, 153	C_25_ H_48_ O_12_P^−^	1.69	PI (16:0/0:0)
36	12.54	721.5034	675, 415, 397, 277	C_45_ H_69_O_7_^−^	0.5	DGMG 18:3*
37	16.99	714.5114	279, 255	C_39_ H_73_NO_8_P^−^	6.3	PE (16:0/18:2)
38	11.18	227.1278	183, 165	C_12_H_19_O_4_^−^	0.025	Traumatic acid
39	13.10	312.3250	294, 102, 88, 57	C_20_H_42_NO^+^	1.51	Eicosanamide
40	14.79	228.2314	102, 88, 57	C_14_H_30_NO^+^	3.33	Tetradecanamide
41	15.17	242.2470	102, 88, 57	C_15_H_32_NO^+^	3.51	Pentadecanamide
42	15.57	256.2624	238, 102, 88, 57	C_16_H_34_NO^+^	4.18	Hexadecanamide
43	15.73	284.2938	266, 102, 88, 57	C_18_H_38_NO^+^	3.31	Octadecanamide
*Flavonoids*						
44	6.78	609.1460	343, 301	C_27_H_29_O_16_^−^	1.7	Quercetin-3-rutinoside (Rutin)
45	7.13	463.0867	301, 271, 255	C_21_H_19_O_12_^−^	0.93	Quercetin-3-O-glucoside (Isoquercetrin)
46	7.16	593.1519	284	C_27_H_29_O_15_^−^	2.9	Kaempferol-3-O-rutinoside
47	7.34	505.0987	301, 283, 255	C_23_H_21_O_13_^−^	1.02	Quercetin-3-O-acetylhexoside
48	7.38	551.1048	463, 303	C_24_H_23_O_15_^+^	3.1	Quercetin-3-O-malonylglucoside
49	7.40	433.0775	301, 271, 255	C_20_H_17_O_11_^−^	2.18	Quercetin-3-O-pentoside
50	7.48	623.1604	314, 427	C_28_H_31_O_16_^−^	0. 41	Isorhamnetin-3-O-rutinoside
51	7.66	447.0931	284	C_21_H_19_O_11_^−^	2.1	Kaempferol-4'-O-glucoside
52	7.79	477.1036	314, 385, 271	C_22_H_21_O_12_^−^	1.6	Isorhamnetin-3-O-glucoside
53	9.44	285.0404	133, 151	C_15_H_9_O_6_^−^	3.2	Luteolin
54	9.47	301.0351	151, 179, 121	C_15_H_10_O_7_^−^	2.8	Quercetin
55	10.71	285.0405	239, 143	C_15_H_9_O_6_^−^	4.1	Kaempferol
56	10.93	315.0508	300, 151, 107	C_16_H_11_O_7_^−^	3.6	Isorhamnetin
*Phenolics*						
57	1.89	191.0555	127, 111, 87, 85	C_7_H_11_O_6_^−^	2.5	Quinic acid
58	3.75	153.0186	109	C_7_H_5_O_4_^−^	2.3	Protocatehuic acid
59	4.93	353.0874	191, 173, 135	C_16_H_17_O_9_^−^	1.8	3-CQA (neochlorogenic acid)
60	5.29	477.1609	293, 183, 131	C_20_ H_29_O_13_^−^	1.3	Kelampayoside A
61	5.36	179.0341	135	C_9_H_7_O_4_^−^	1.3	Caffeic acid
62	5.44	487.1447	193, 149	C_21_H_27_O_13_^−^	0.2	Pentosylhexoxyl Ferulic acid
63	5.86	337.214	191, 173	C_16_H_17_O_8_^−^	6.6	Coumaroyl quinic acid
64	6.15	367.127	191, 193, 173	C_17_H_19_O_9_^−^	0.5	5-Feruloylquinic acid
65	6.19	371.0984	249, 121	C_16_H_19_O_10_^−^	3	Benzoyl glucuronosyl glycerol
66	6.39	313.0718	269, 159, 109	C_17_ H_13_O_6_^−^	3.75	*E*-3-(3,4-dihydroxybenzylidene)-5-(3,4-dihydroxyphenyl)dihydrofuran-2-one
67	6.80	537.1026	493, 295	C_27_H_21_O_12_^−^	0.2	Lithospermic acid
68	7.44	515.1182	353, 191, 179	C_25_H_23_O_12_^−^	7.44	1,5‐DCQA (1,5-Dicaffeoylquinic acid)
69	7.61	353.08694	191, 135 179	C_16_H_17_O_9_^−^	0.7	5-CQA (Chlorogenic acid)
70	7.73	598.2485	419, 401, 383, 371, 265, 205	C_28_H_40_O_13_N^+^	1.5	Syringaresinol-O-*β*-D-glucopyranoside**
71	7.83	353.0868	191, 179, 173, 135	C_16_ H_17_ O_9_^−^	0.32	4-CQA (Cryptochlorogenic acid)
72	7.93	359.072	197, 161	C_18_H_15_O_8_^−^	2.2	Rosmarinic acid
73	7.99	493.1138	313, 295	C_26_H_21_O_10_^−^	0.4	Salvianolic acid A
74	8. 42	717.1445	519, 339, 321, 295	C_36_ H_29_ O_16_^−^	0.75	Salvianolic acid B
75	8.60	499.126	353, 337, 179, 173, 191	C_25_H_23_O_11_^−^	0.26	3-*P*-Coumaroyl-4-caffeoylquinic acid
76	8.86	529.135	191, 179, 173, 161, 135	C_26_H_25_O_12_^−^	0.7	Feruloyl caffeoyl quinic acid
77	9.47	677.1502	515. 353, 191	C_34_H_29_O_15_^−^	0.256	Tricaffeoyl quinic acid
78	9.68	491.0982	311, 293	C_26_H_19_O_10_^−^	1.85	Salvianolic acid C
79	9.76	543.1521	367, 349, 193	C_27_H_27_O_12_^−^	4.41	Diferuloyl quinic acid
*Coumarin*						
80	5.33	177.0185	149, 133, 105	C_9_H_5_O_4_^−^	1.21	Esculetin
81	6.96	193.04901	137, 133, 122	C_10_H_9_O_4_^+^	2.72	Scopoletin
*Lignans*						
82	9.40	519.1868	357, 173	C_26_ H_31_O_11_^−^	1.4	Styraxlignolide E
*Sugars*						
83	0.82	341.1084	179, 161	C_12_ H_21_O_11_^−^	1.6	Sucrose

*Indicates the [M + HCOH]^–^ adduct; **Indicates the [M + NH_4_]^–^ adduct. Chemical structures of some main compounds were presented in Fig. 4S

### Characterization of pyrolizidine alkaloids

**Fig. 2:**
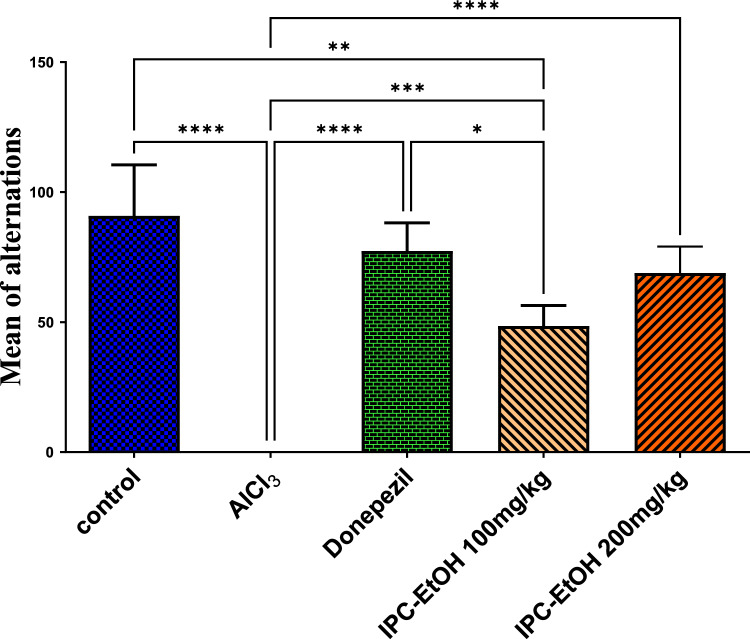
Results are expressed as means of % of alternations ± SE, *n* = 5. Significance Data are expressed as ± SD. Statistical analysis was performed using the one-way analysis of variance (ANOVA) followed by the Tukey’s multiple comparison test, *p ≤ 0.05, **p ≤ *0.01*, ***p ≤ 0.001, ****p ≤ 0.0001, where AlCl_3_ is the positive control group

**Fig. 3:**
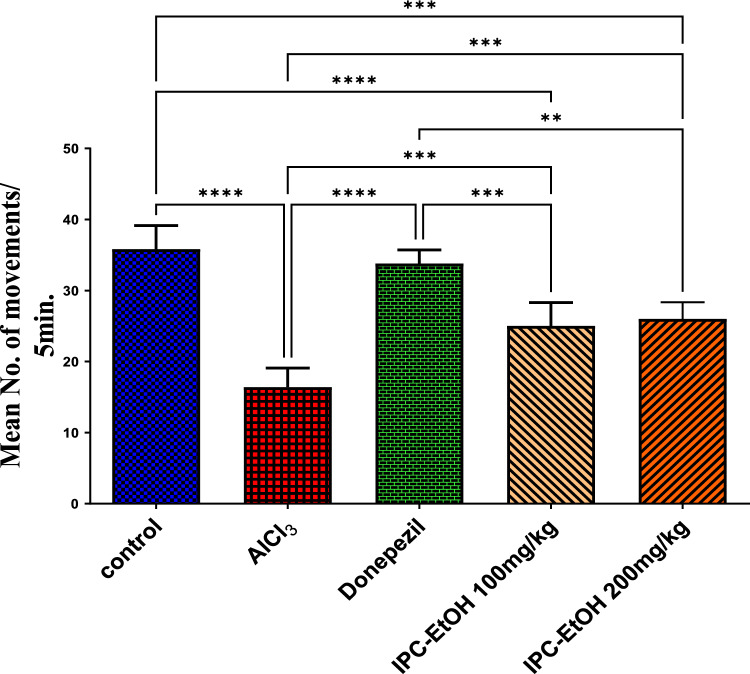
Results are expressed as means of movement/5 min ± SE, *n* = 5. Significance Data are expressed as ± SD. Statistical analysis was performed using the one-way analysis of variance (ANOVA) followed by the Tukey’s multiple comparison test, **p ≤ *0.01*, ***p ≤ 0.001, ****p ≤ 0.0001, where AlCl_3_ is the positive control group

The UPLC-ESI–MS based molecular network helped in annotation of several pyrrolizidine alkaloids (PAs) from the positive ionization mode (Fig. 1A and B). These alkaloids are a class of ornithine-derived alkaloids that are present in some plant species and insects that consume them to protect themselves from predators (Hartmann 1999; Moreira et al. 2018). They are typically found as pyrrolizidine alkaloids *N*-oxides or tertiary bases (Fig. 2S) (Moreira et al. 2018). They are generally found as esters made up of at least one necic acid and a necine base (Wei et al. 2021; Moreira et al. 2018). The necine base is made up of pyrrolizidine, an amino alcohol constructed of double five-membered rings joined together by a nitrogen atom (Jayawickreme et al. 2023; Moreira et al. 2018). PAs are categorized into three groups established on the necine bases: retronecine (seven stereoisomers), otonecine, and platynecine types (Fig. 3S) (Li et al. 2008; Wei et al. 2021). They differ in the double-bond saturation and the ring cyclicity that is interrelated to their poisonousness (Schramm et al. 2019). Nearly all necines have a hydroxymethyl group at C-1, and they typically also have a hydroxyl group at C-7, where esterification takes place. Furthermore, the necine may have one or two hydroxyl groups at C-2 or C-6, which could lead to the creation of stereoisomers (Roeder 2000; Schramm et al. 2019; Products 2014).

**Fig. 4:**
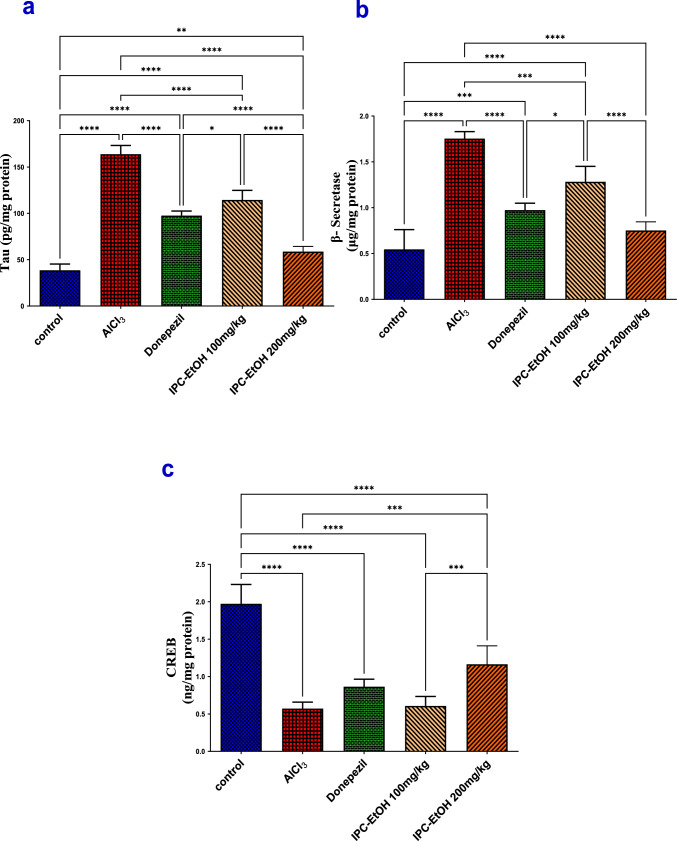
The hippocampal levels of (**a**) tau, (**b**) β-secretase, and (C) CREB in different treated groups compared to AlCl_3_ model group. Results are expressed as means ± SD, *n* = 5, where statistical analysis was performed using the one-way analysis of variance (ANOVA) followed by the Tukey’s multiple comparison test. *p ≤ 0.05, **p ≤ 0.01, ***p ≤ 0.001, ****p ≤ 0.0001

Several PAs were detected by MS spectrum analysis, which was indicated by their even high-resolution masses and significantly stronger response in positive ionization mode. Three unsaturated PAs, **1, 7,** and** 16,** exhibited molecular ions [M + H]^+^ at *m/z* 300.1802, 314.1951, and 342.1901, consistent with formulas C_15_H_26_NO_5_^+^, C_16_H_28_NO_5_^+^, and C_17_H_28_NO_6_^+^, respectively. The product ions C_8_H_12_NO^+^ at *m/z* 138, C_8_H_10_N^+^ at *m/z* 120, and C_6_H_8_N^+^ at *m/z* 94 are distinctive of the retronecine type (Fig. 4S) (Li et al. 2008; These et al. 2013; Xiong et al. 2014; Lu et al. 2021; Stanoeva et al. 2022). The free hydroxyl group at C-7 was verified through the base peak at *m/z* 138 [M-C_7_H_13_NO_4_]^+^, rising from breaking the weak allylic ester link at C-9 (El-Shazly 2004; Pedersen and Larsen 1970; El-Shazly et al. 1996). In addition to the complete similarity of fragments of compounds **16** and **1**, the difference between the protonated masses of the two compounds was 42 mass units. Consequently, compound **16** has an additional acetyl group at C-9 hydroxyl, as it loses an acetic acid molecule to form [M + H-CH_3_COOH]^+^ and form a fragment ion at *m/z* 300, and the acetylation undergoes C-3` in prior investigations (Hartmann and Witte 1995; Tei 2000; El-Shazly 2004).

Another two unsaturated PAs, **5** and 1**2,** showed lower [M + H]^+^ by 16 amu than compounds **1** and** 7** due to the absence of the hydroxyl group at C-7. Their protonated masses are 284.1845 and 298.2004, they predicted molecular formulae C_15_H_26_NO_4_^+^ and C_16_H_28_NO_4_^+^, respectively. They produced fragment ions at *m/z* 140 and 122, the base peak at *m/z* 122 [M-C_7_H_13_O_4_]^+^, results from cleavage of the weak allylic ester bond at C-9, which confirms the skeleton related to supinidine necine esterified at C-9 (Prada et al. 2020).

A series of eight saturated PAs have been noticed in the chromatogram, their fragment ions illustrate the two types of saturated PAs (platynecine and trachelanthamidine) (Lu et al. 2021).

Compounds **4**, **11**, **14**, and **17** exhibited [M + H]^+^ at *m/z* 286.2004, 300.2159, 328.2108, and 342.2266, respectively, consistent with their molecular formulas (C_15_H_28_NO_4_^+^), (C_16_H_30_NO_4_^+^), (C_17_H_30_NO_5_^+^), and (C_18_H_32_NO_5_^+^), respectively. They represent distinctive fragment ions at *m/z* 124 and 142 representing platynecine monoester (saturated retronecine) (Li et al. 2008; Xiong et al. 2014; Lu et al. 2021). In addition, PAs **6**, **13**, **15**, and **18** are related to trachelanthamidine (saturated supinidine), as their protonated molecular ions showed *m/z* at 302.1951(C_16_H_28_NO_5_^+^), 316.2110 (C_16_H_30_NO_5_^+^), 344.2058 (C_17_H_30_NO_6_^+^), and 358.2214 (C_18_H_32_NO_6_^+^), respectively. They produced two identified fragments at *m/z* 140 and 122, which were predictable for the trachelanthamidine pattern (Lu et al. 2021).

Compounds **2** and **9** showed mass spectrum with precursor ions [M + H]^+^ at *m/z* 316.1744 and 330.1899, producing product ions [M + H-18]^+^ at *m/z* 298 and 312, respectively, in addition to two product ions at *m/z* 172 and 138 representing fragmentation of crotanecine skeleton, which is 6,7-dihydroxylated PAs (Singh and Vellapandian 2023). Furthermore, four compounds, **3**, **8**, **10,** and **19**, related to crotanecine appeared in the chromatogram representing parent molecular ions at *m/z* 318.1900, 332.2056, 360.2006 and 374.2163, respectively consistent with their molecular formulae. They represent fragment ions at *m/z* 174 and 156, which are related to the crotanecine pattern (Etienne et al. 2021; Singh and Vellapandian 2023), with a respectable appearance of two fragment ions at *m/z* 300 and 314 for compounds **10** and **19** due to losing an acetic acid molecule to form [M + H-CH₃COOH]⁺, which confirms acetylation at C-3` (El-Shazly 2004).

### Characterization of the other nitrogenous compounds

Adenosine derivatives **23**, **25,** and **27** predicted protonated molecular weights at *m/z* 384.1138, 413.1402, and 314.0901, respectively, they lose their pentosyl group easily, producing specific ion fragments [M + H-C_5_H_9_O_4_]^+^ at *m/z* 182, 252, and 281, respectively. In addition to the distinctive fragment ion of *m/z* 136 relative to adenine, which is the fragment base for adenosine **24** itself (Singh et al. 2021a).

Compounds **21, 22, 26, and 28** showed [M-H]^−^ at *m/z* 243.0617, 290.0883, 203.0817, and 206.0822, consistent with molecular formulas (C_11_H_16_NO_8_^−^), (C_9_H_11_N_2_O_6_^−^), (C_11_H_11_N_2_O_2_^−^), (C_11_H_11_N_2_O_2_^−^), and (C_11_H_12_NO_3_^−^), respectively. These compounds were tentatively identified as arabinosyl uracil, N-fructosyl pyroglutamate, tryptophan, and N-acetylphenylalanine, respectively. On the other hand, an ion [M + H]^+^ at *m/z* 314.1378 for compound **29** was observed that produced product ions at *m/z* 177, 145, and 121 relative to feruloyltyramine. Compound **20** was identified as N-acetyl glutamic acid, it has the molecular formula C_7_H_10_NO_5_^−^, and showed fragment ions at *m/z* 128 and 102 (Singh et al. 2021a).

### Characterization of glycerophospholipids and fatty acids

Glycerophospholipids are lipids with a glycerol backbone that bind to fatty acids in the sn-1 and/or sn-2 positions. Polar phosphor groups in the sn-3 position were used to distinguish between different types of glycerophospholipids (Fang and Barcelona 1998; McDowell et al. 2014). Phospholipids are crucial molecules in cellular membranes and are involved in membrane proteins, receptors, enzymes, and ion channels. Changes in brain phospholipid levels can lead to pathogenic processes. Mass spectrometry imaging has enabled precise regional phospholipid distribution. Understanding changes in brain phospholipid levels during aging is essential to differentiate between pathogenic and normal processes (Jin et al. 2020). It has been reported that Alzheimer’s disease (AD) lowers phospholipid levels (Haque et al. 2023).

Most lipids, phosphatidylglycerol (PG), phosphatidylcholine (PC), phosphatidylinositol (PI), and phosphatidylethanolamine (PE) decrease slowly with age, with less than 10% loss between 40 and 100 (Kosicek and Hecimovic 2013).

Two compounds, **33** and** 34,** showed [M + H]^+^ at *m/z* 496.3386 and 758.5667, respectively, the molecular formula of these compounds could be calculated as C_24_H_52_NO_7_P and C_42_H_81_NO_8_P, respectively, within the mass errors of 2.31 and 2.6 ppm. The loss of the choline moiety [C_5_H_14_NO_4_P + H]^+^, resulted in the appearance of a product ion at *m/z* 184.0737, and they were deduced to be PC (16:0/0:0) and PC (16:0/18:2), respectively (Jin et al. 2020).

Four compounds (**30–32,** and** 37),** illustrated deprotonated molecular ions at *m/z* 721.5028, 483.2726 and 745.5028, and 714.5114 for PG (16:0/16:0), PG (16:0/0:0), PG (16:0/18:2), and PE (16:0/18:2), respectively. MS^2^ spectrum of these metabolites showed a product ion at *m/z* 255.2 attributed to palmitic acid (16:0), additionally, a product ion at *m/z* 279.2 characterized to linoleic acid (18:2) appeared in **32** and **37** (Calvano et al. 2020).

Compound **36** digalactosylmonoacylglycerol (DGMG) has been detected in the LC–MS spectrum. It is formed of a hydrophobic molecule of linolenic acid linked to the glycerol moiety and to a hydrophilic molecule of digalactose. It was annotated in the chromatogram as precursor type [M + HCOO]^−^ of *m/z* 721.5034, representing the molecular formula C_45_H_69_O_7_. It displayed MS^2^ fragmentation ions at m/z 675 [M-HCOO-H]^−^, 415, and 277 resulting from the cleavage at the C-1 position and loss of linolenic acid [M-C_18_H_29_O_2_]^−^ (Zannella et al. 2021).

Compound **35** showed characteristic fragments at *m*/*z* 315 [glycerophosphoinositol]^−^,* m*/*z* 255 [palmitic acid]^−^, *m*/*z* 241 [M-palmatic acid-glycerol]^−^, *m*/*z* 153 [M-linoleic acid-inositol]^−^, and *m*/*z* 79 [PO_3_]^−^, resulting from the deprotonated molecular ion peak of 571.2888 and was annotated as PI (16:0/0:0) (Calvano et al. 2020). Compound **39** was identified as traumatic acid based on the fragment ion at *m/z* 183 [M-H-CO_2_]^−^ (Klamrak et al. 2023).

Five amino fatty acids, **39–43**, were tentatively identified at *m/z* 312.3250 (C_20_H_42_NO^+^), 228.2314 (C_14_H_30_NO^+^), 242.2470 (C_15_H_32_NO^+^), 256.2624 (C_16_H_34_NO^+^), and 284.2938 (C_18_H_38_NO^+^). Amino fatty acids are characterized by the loss of H_2_O and cleavage of the carbon chain in their MS/MS fragment ions. These fragment ions can be broken into fragments at *m/z* 102, 57, or 106 and further dehydrated to produce fragment ions at *m/z* 88 or 70 (Zhang et al. 2022).

### Characterization of flavonoids

The molecular family of flavonoids extracted from the molecular network of negative mode (Fig. 1C) revealed 3 flavonol diglycosides, **44, 46,** and **50,** with molecular ions at *m/z* 609.1460, 593.1519, and 623.1604, producing distinctive daughter ions at *m/z* 301, 285, and 315, respectively, due to the loss of rhamnose and glucose moieties, and were identified as quercetin-3-*O*-rutinoside (rutin), isorhamnetin-3-O-rutinoside, and kaempferol 3-O-rutinoside, which is in accordance with the literature data (Mateos et al. 2018; Kramberger et al. 2020; Xu et al. 2022).

It is possible to identify Compound **47** as quercetin-3-O-acetylhexoside because it exhibits a deprotonated molecular ion at *m/z* 505.0987. The MS^2^ fragmentation ion produced a base signal at *m/z* 301 (quercetin) that was accompanied by the fragment ion at *m/z* 463, which resulted from the loss of the 42-amu fragment corresponding to the acetyl radical (Garcia et al. 2025). Compound **48** was identified as quercetin-3-O-malonylglucoside with a deprotonated molecular ion peak at 551.1048, consistent with its formula C_24_H_23_O_15_^+^, which was confirmed with MS fragmentation by displaying an ion fragment at *m/z* 463 due to decarboxylation of its malonyl unit, then the appearance of an ion fragment at *m/z* 303 corresponding to quercetin (Thabti et al. 2012; Escobar-Avello et al. 2021).

Compounds **45**, **49**, **51**, and **52** have been identified in the flavonoid family network (Fig. 1C) as quercetin-3-O-glucoside**,** quercetin-3-O-pentoside, kaempferol-4ʹ-glucoside**,** and isorhamnetin-3-O-hexoside, respectively, by comparing their molecular weights and fragment ions to those reported previously (Escobar-Avello et al. 2021). In addition, four aglycones, quercetin **53**, luteolin **54**, kaempferol **55,** and isorhamnetin **56,** have also been identified (Ruan et al. 2019).

### Characterization of phenolic compounds

MS/MS spectrum displayed three mono-caffeoylquinic acid isomers, **59**, **69,** and **71,** with a base peak ion at *m/z* 353 [M-H]^−^ and similar secondary ions at *m/z* 191 [quinic-H]^−^, 179 [caffeoyl-H]^−^, 161 [caffeoyl-H-H_2_O]^−^, 135 [caffeoy-H-CO_2_]^−^, and 173 [quinic-H-H_2_O]^−^, these isomers can be differentiated in the negative-ion mode based on the relative abundances of major product ions (Willems et al. 2016). The base peak of 3-CQA was at *m/z* 191 resulting from quinic acid, while ion peaks from caffeoyl and quinic acid groups were not significant. Whereas in 5-CQA, not only the characteristic fragment ions of *m/z* 191 but also the strong caffeoyl characterization fragment ions at m/z 179 and 135. For 4-CQA, the base peak was at *m/z* 173, with a strong appearance to the other fragments (Ruan et al. 2019).

Another seven derivatives of caffeoylquinic acid, **63**, **64**, **68**, **75**, **76**, **77**, and **79,** have been detected and tentatively identified, representing deprotonated molecular ions and fragments consistent with those reported in previous studies (Clifford et al. 2005; Ruan et al. 2019; Nengovhela et al. 2022). Several phenolic acids, quinic acid **57**, protocatechuic acid **58**, caffeic acid **61**, lithospermic acid **67**, and rosmarinic acid **72,** were recognized by comparing their fragmentation ions with those published (Damašius et al. 2014; Ruan et al. 2019; Singh et al. 2021a; Zhu et al. 2022; Pan et al. 2024). Compound **66** exhibited a peak at *m/z* 313.0718 [M-H]^−^, which assigned the deprotonated molecular formula C_17_H_13_O_6_^−^. It produced fragment ions at *m/z* 269 (carboxylic group loss) and two fragments at *m/z* 159 and 109 due to cleavage of bonds at C-1′/C-5 and C-7′/C-1′ (Bader et al. 2011).

MS/MS spectral analysis revealed the detection of three salvianolic acid derivatives, salvianolic acid A **73**, salvianolic acid B **74**, and salvianolic acid C **78**. They have precursor ions at *m/z* 493.1138, 717.1445, and 491.0982, respectively, producing daughter ions at *m/z* 295, 519, and 293, respectively, due to loss of danshensu [M-H-198]^−^, in addition to fragment ions at *m/z* 313 and 311 for salvianolic acid A and salvianolic acid, from the loss of caffeic acid [M-H-180]^−^ (Liu et al. 2007; Cheng et al. 2019; Zhu et al. 2022). The molecular network of the negative mode (Fig. 1C) showed a family of several nodes related to salvianolic acid B with mass difference – 16 amu and + 30 amu which could be identified as deoxygenated and methoxylated salvianolic acid B, respectively. As well as this cluster indicated the node at *m/z* 551.1190 as methyl melitrate. In addition, 2 ferulic acid glycoside derivatives, **62** and **80,** showed molecular ion peaks at 487.1447 and 679.2980, indicating perfect fit fragment ions of ferulic acid at *m/z* 193 [M-H-132(pent)-162(hex)]^−^ and 193 [M-H-486(3hex)]^−^, respectively (Abdel Ghani et al. 2023; Babacan et al. 2023).

Compound **70** generated [M + NH_4_]^+^ ion at *m/z* 598.248 (C_28_H_40_O_13_N^+^), producing a series of ions at *m/z* 419 [M + NH_4_-OGlu]^+^, 401 [M + NH_4_-OGlu-H_2_O]^+^, 383 [M + NH_4_-OGlu-2H_2_O]^+^, 371 [M + NH_4_-OGlu-H_2_O-2CH_3_]^+^, and 265 [M + NH_4_-C_14_H_19_O_8_]^+^. It was deduced to be syringaresinol-O-*β*-D-glucopyranoside based on the data from the literature (Jiao et al. 2018).

The high-collision energy spectrum revealed the presence of a phenylpropanoid, kelampayoside A (**60)**, with a precursor ion peak at *m/z* 477.1609. It produced fragment ions at *m/z* 293 (C_11_H_17_O_9_), 183 (C_9_H_11_O_4_) and 131 (C_7_H_5_O_2_) (Essono Mintsa et al. 2023). The spectrum also illustrated characteristic [M-H]^−^ at *m/z* 371.0984 for compound **65**, which produced MS^2^ fragments at *m/z* 121 and 249 due to the loss of benzoic acid (122 Da) to give *m/z* 249, and it was identified as benzoyl glucuronosyl glycerol (Cai et al. 2011a; Stark et al. 2017).

### Characterization of coumarins

Compound **80** was tentatively assigned as esculetin; it displayed a parent peak [M-H]^−^ at *m/z* 177.01840 and product ions at 149 [M-H–CO]^−^, 121 [M-H-2CO]^−^, and 133 [M-H-CO_2_]^−^ (Ruan et al. 2019; Singh et al. 2021a). On the other hand, compound **82** displayed [M + H]^+^ at *m/z* 193.04901 with a molecular formula of C_10_H_9_O_4_^+^ and product ions at 137, 133, and 122, which suggested that compound **81** was scopoletin (Zeng et al. 2015).

Negative electrospray ionization revealed one bioactive lignin metabolite **82**, producing [M-H]^−^ at *m/z* 519.1868. It produced a significant product ion at *m/z* 357, which represents the cleavage of the hexosyl moiety [M-H-162]^−^, and was tentatively identified as styraxlignolide E (Min et al. 2004; Ji et al. 2011). Compound **83** [M-H]^−^ at *m/z* 341.1084, equivalent to two ester bond hexoses, generated fragment ions at *m/z* 179 [M-H-162]^−^, resulting from the loss of a hexosyl moiety, was deduced to be sucrose (Babacan et al. 2023).

### Behavioral stress tests

#### T- maze test results

In the current study, oral administration of AlCl_3_ for four successive weeks resulted in significantly (*p* < *0.0001*) severe deterioration in cognition, manifested by absence of alternation of rats in the T-maze test. On the other hand, treatment of rats with donepzil (0.75 mg/kg), and both extracts in low and high doses given together with AlCl_3_ for 4 successive weeks significantly protected against cognitive deterioration, observed by significant difference from the positive control group with the highest effect of donepezil and the high dose of IPC-EtOH (200 mg/kg), as they did not show any significant difference from the negative control group. While the administration of IPC-EtOH at a dose of 100 mg/kg could significantly (*p* = 0.0005) protect against cognitive deterioration but their cognitive function did not return to the normal levels and remained significant (*p* = 0.0020) and (*p* = 0.0489) from the normal control group and donepezil significantly. Results are expressed in Fig. 2.

### The open field test results

In the current study, oral administration of AlCl_3_ for four successive weeks resulted in significantly (*p* < 0.0001) severe deterioration in psychological state, manifested by significant reduction in number of movements in the open field arena for 5-min duration. Treatment of rats with donepezil used as a standard drug (0.75 mg/kg) concomitant with AlCl_3_ significantly (*p* < 0.0001) improved the psychological state compared to the positive control group. Also, treatment with both doses of the extract significantly improved the psychological state of rats compared to the positive control group manifested by a significant (*p* ≤ 0.001) increase in the number of movements. Yet, this effect is significantly (*p* = 0.0002) and (*p* < 0.0001) less than the negative for IPC-EtOH 100 mg/kg and 200 mg/kg, respectively, and also still significant from standard groups manifested by significantly (*p* = 0.0006) and (*p* = 0.0023) less number of movements for IPC-EtOH 100 mg/kg and 200 mg/kg respectively. Results are expressed in Fig. 3.

### Biochemical results

#### *IPC-EtOH extract alleviates AlCl*_*3*_*-induced changes in tau, β-secretase and, CREB hippocampal levels*

In the current study, oral administration of AlCl_3_ for 4 successive weeks resulted in a prominent elevation (*p* < 0.0001) in the hippocampal levels of tau (163.80 ± 4.26) and β-secretase (1.75 ± 0.03), the hallmarks of AD, with a significant (*p* < 0.0001) downregulation of CREB (0.57 ± 0.04) in comparison with the normal group (38.70 ± 2.97, 0.55 ± 0.1, 1.97 ± 0.12) for tau, β-secretase, and CREB, respectively. This was ameliorated by IPC-EtOH (100 mg/kg/day) producing a notable decrease in the levels of tau (*p* < 0.0001) and β-secretase (*p* = 0.0002) by 127.4% and 86.2%, respectively, as compared to the AlCl_3_ group, and this effect was analogous to that produced by donepezil, where it provokes a significant (*p* < 0.0001) decrease in tau and β-secretase by 171.1% and 142.8%, respectively, as compared to the AlCl_3_ group, but could not elicit any significant changes in CREB by both IPC-EtOH and donepezil (Fig. 4).

While in the group administered IPC-EtOH (200 mg/kg/day), the significant declines were recorded in tau and β-secretase by 271.5% and 183.4%, respectively, as compared to the AlCl_3_ group and provoked a notable (*p* = 0.0004) elevation in CREB by 30% as compared to the AlCl_3_ group (Fig. 4).

### *IPC-EtOH extract alleviates AlCl*_*3*_*-induced oxidative stress in hippocampi*

**Fig. 5:**
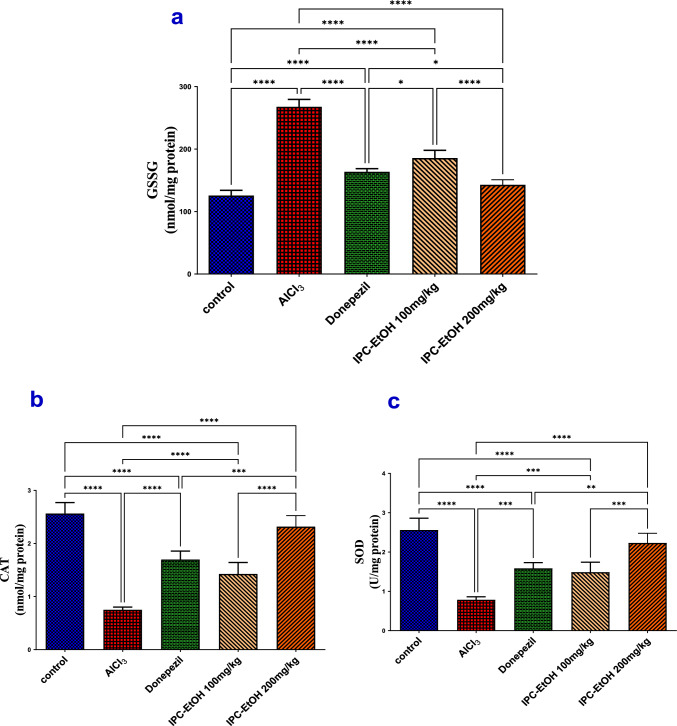
The hippocampal levels of (**a**) GSSG, **b** CAT, and **c** SOD in different treated groups compared to the AlCl_3_ model group. Results are expressed as means ± SE, *n* = 5 Statistical analysis was performed using the one-way analysis of variance (ANOVA) followed by the Tukey’s multiple comparison test. *p ≤ 0.05, **p ≤ 0.01, ***p ≤ 0.001, ****p ≤ 0.0001

The level of GSSG in the hippocampi of the AlCl_3_ group was significantly (*p* < 0.0001) upsurged by 113.1%. This was associated with a profound (*p* < 0.0001) reduction in the hippocampal level of CAT and SOD by 3.4 and 3.3 folds as compared to the NE group. The administration of IPC-EtOH extract at dose levels (100 or 200 mg/kg) in AlCl_3_-subjected rats could significantly (*p* < 0.0001) diminish the elevated GSSG by 65.3% and 99.4% as compared to the AlCl_3_ group **(**Fig. 5a**)**. These replenished effects in the hippocampal level of GSSG with a consistent (*p* < 0.0001) elevation of CAT and SOD hippocampal levels by 26.3% and 27.4%, respectively, in the administration of IPC-EtOH extract (100 mg/kg) and by 61.1% and 56.5%, respectively, in the administration of IPC-EtOH extract (200 mg/kg), as compared to the AlCl_3_ group, producing a comparable effect with that of the donepezil group **(**Fig. 5b and c**)**.

### *IPC-EtOH extract modulates AlCl*_*3*_* induced changes in GSK-3β, and FOXO3a hippocampal content*

**Fig. 6:**
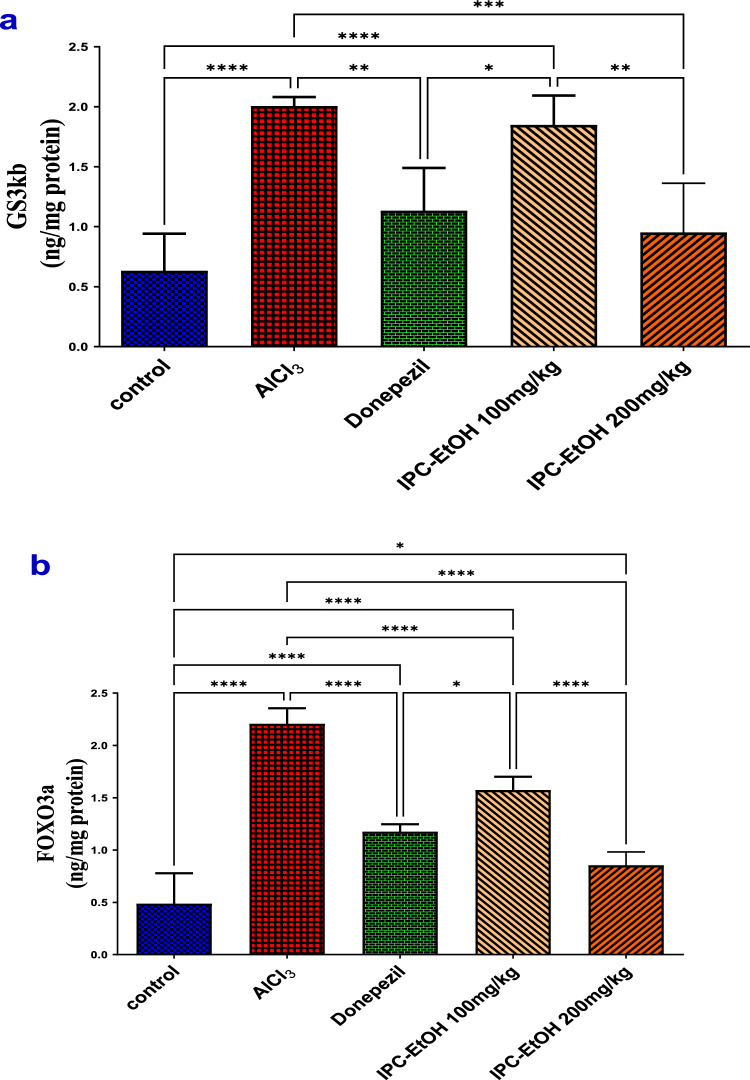
The hippocampal levels of (**a**) GS3kb and **b** FOXO3a in different treated groups compared to the AlCl_3_ model group. Results are expressed as means ± SD, *n* = 5. Statistical analysis was performed using the one-way analysis of variance (ANOVA) followed by the Tukey’s multiple comparison test, *p ≤ 0.05, **p ≤ 0.01, ***p ≤ 0.001, ****p ≤ 0.0001

GSK-3β and FOXO3a concentrations were significantly (*p* < *0.0001*) upregulated in the hippocampi of AlCl_3_ rats by 3.2- and 4.5-fold respectively, as compared to NE rats. Such effects were mitigated by treating the AlCl_3_ rats with IPC-EtOH at a dose of 200 mg/kg, resulting in a prominent reduction in their concentration by 166.5% (*p* = 0.0002) and 278.4% (*p* < *0.0001*) for GSK-3β and FOXO3a, respectively, as compared to the AlCl_3_ group. Similarly, the reference drug donepezil significantly down-regulated the level of GSK-3β (*p* = 0.0016) and FOXO3a (*p* < 0.0001) by 137.9% and 212.1%, respectively, as compared to the AlCl_3_ group, producing analogous effects to that of IPC-EtOH extract (200 mg/kg). On the other hand, the low-dose administration of IPC-EtOH at a dose of 100 mg/kg could not elicit any changes in the level of GSK-3β but could produce a notable (*p* < 0.0001) reduction in the level of FOXO3a by 130.5% as compared to the AlCl_3_ group (Fig. 6).

### Results of light microscopy observations

**Fig. 7:**
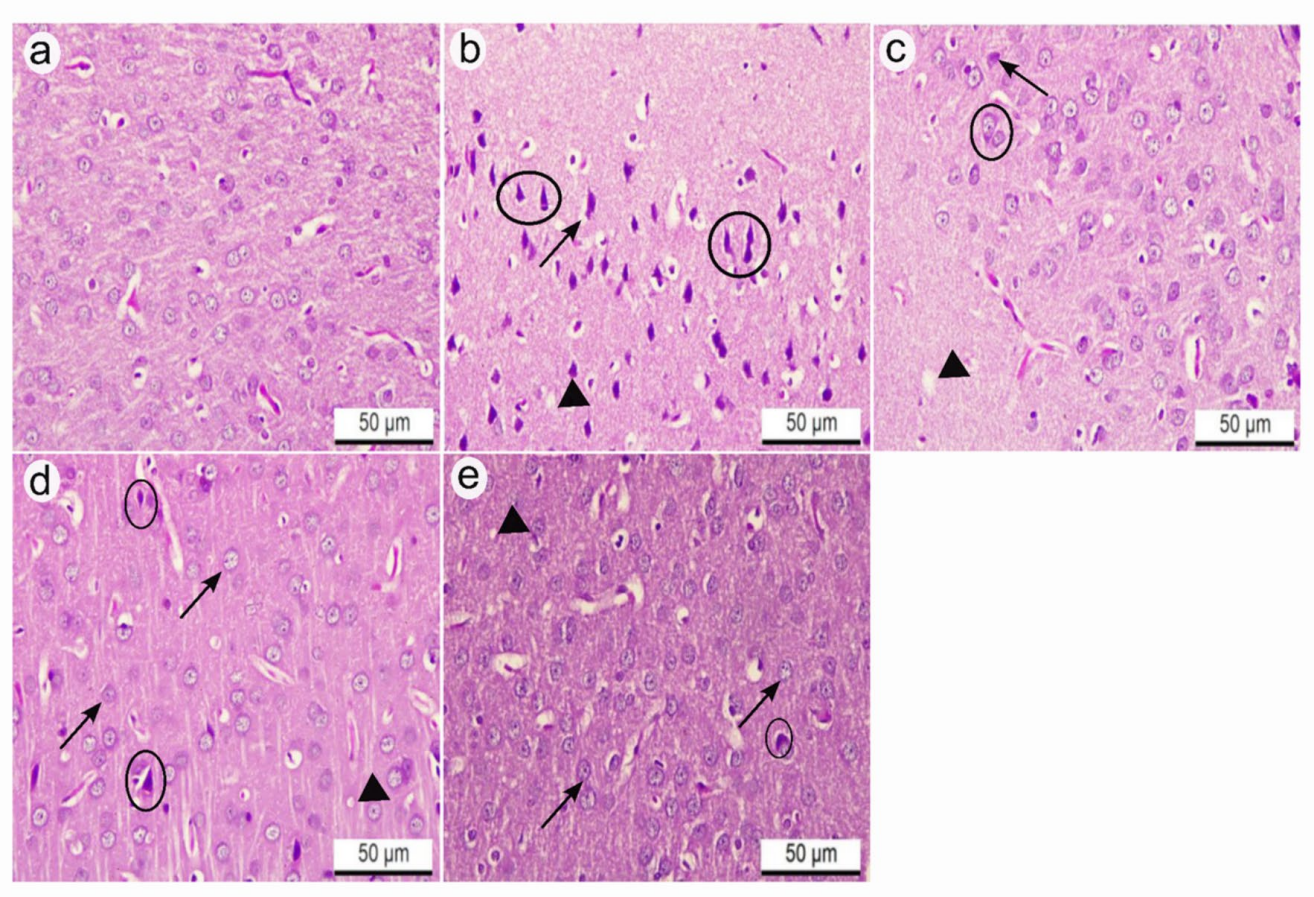
**a**: **e** Cerebral cortex of adult Wistar rats. H&E. X400. **a** Control rats showed normal histoarchitecture of neurons, neuroglia, and neuropil of the cerebral cortex. **b** Rats treated with aluminum chloride (AlCl_3_) revealed shrunken and pyknotic neurons (black circles) with pericellular spaces (black arrow) and vacuolation of the neuropil (black arrowhead). **c** Rats treated with donepezil hydrochloride as a reference drug revealed nearly normal and lightly stained pyramidal neurons (black circle), except for a few degenerated neurons (black arrow) and less neuropil vacuolation (black arrowhead). **d** Rats treated with IPC-EtOH (100 mg/kg) showed nearly normal-shaped neurons (black arrows), few pyknotic neurons with pericellular space (black circles), and diminished neuropil vacuolation (black arrowhead). **e** Rats treated with IPC-EtOH (200 mg/kg) had nearly normal histoarchitecture of cerebral cortex tissue with nearly normal shaped neurons (black arrows), less neuropil vacuolation (black arrowhead) and rarely observed degenerated neurons (black circle)

Histological examination of H&E-stained cerebral cortex sections of control rats showed normal histoarchitecture of neurons, neuroglia, and neuropil (Fig. 7a). Otherwise, cerebral cortex sections of rats treated with aluminum chloride (AlCl_3_) displayed several degenerative changes in the histoarchitecture compared to the control group, such as shrunken and pyknotic neurons with pericellular spaces and vacuolation of the neuropil (Fig. 7b). However, the cerebral cortex of rats treated with donepezil hydrochloride as a reference drug revealed marked maintenance of the nearly normal structure of most neurons that appeared pyramidal and lightly stained, except for a few degenerated neurons and less neuropil vacuolation compared with the AlCl_3_-treated group (Fig. 7c). Meanwhile, the cerebral cortex tissue of rats treated with IPC-EtOH (100 mg/kg) showed nearly normal-shaped neurons, but pyknotic neurons with pericellular space and diminished neuropil vacuolation were observed (Fig. 7d). Contrariwise, rats treated with IPC-EtOH (200 mg/kg) had nearly normal histoarchitecture of cerebral cortex tissue, with preservation of the nearly normal shaped neurons that appeared vesicular with less neuropil vacuolation and rarely observed degenerated neurons compared to AlCl_3_-treated group (Fig. 7e).

**Fig. 8:**
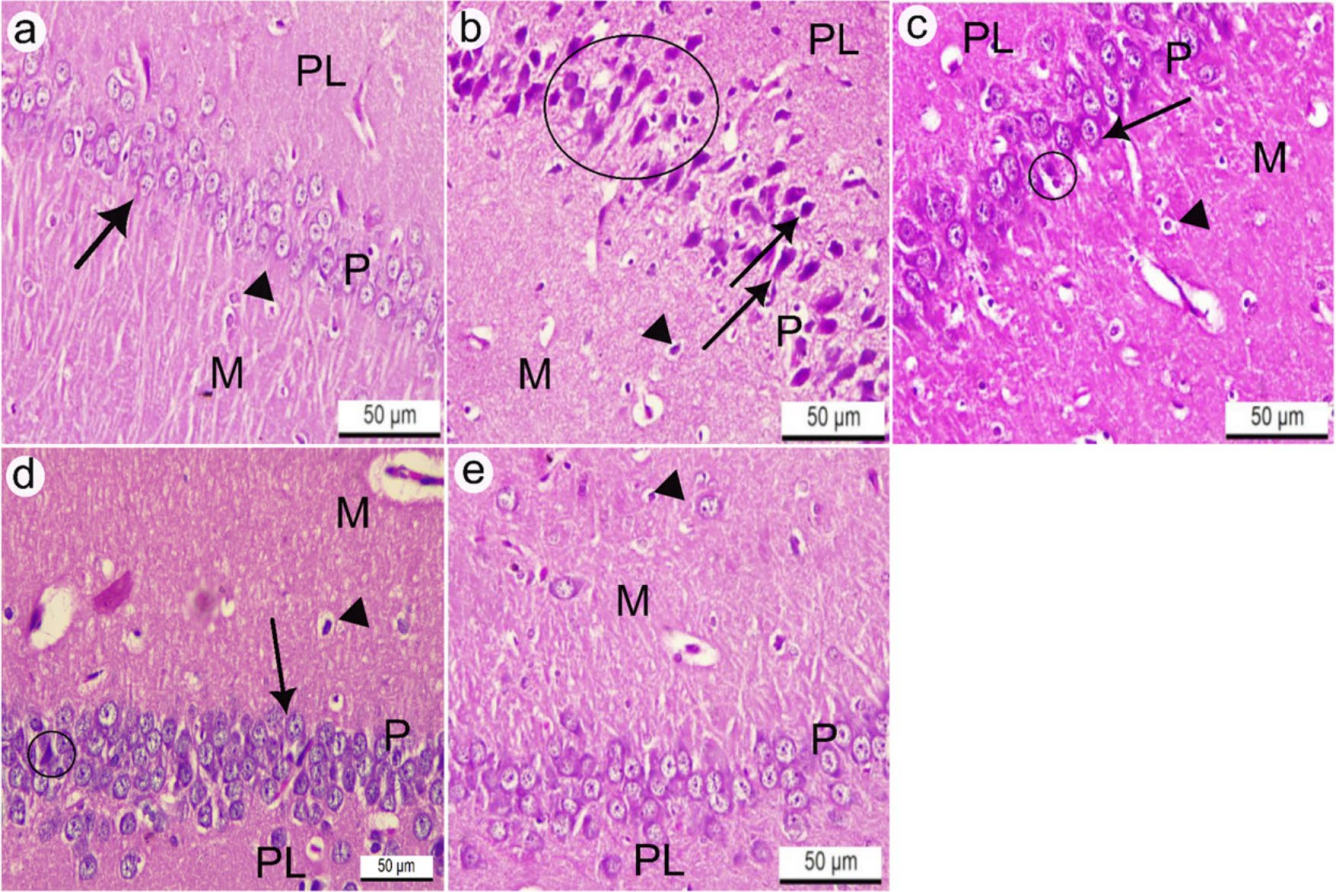
**a**, **e** Hippocampal tissues of adult Wistar rats. H&E.X100. **a** Control rats comprised three normal layers: molecular (M), Purkinje (P), and polymorphic (PL). The Purkinje cell layer consists of triangular shaped neurons with vesicular nuclei (black arrow). **b** Rats treated with AlCl_3_ revealed pyknosis of glial cells with pericellular space (black arrowhead) in the molecular layer (M), disarrangement of Purkinje cells (black circle) that appeared shrunken and pyknotic with perineuronal space (black arrow). **c** The hippocampal tissue of rats treated with donepezil hydrochloride as a reference drug revealed the maintenance of nearly normally arranged Purkinje cells (black arrow) that appeared triangular and had vesicular nuclei, except for a few pyknotic Purkinje cells (black circle) and glial cells with pericellular spaces (black arrowhead). **d** Rats treated with IPC-EtOH (100 mg/kg) showed early normal arranged Purkinje cells (black arrow), but there were few degenerated Purkinje cells (black circle) and pyknotic glial cells (black arrowhead) in the molecular layer (M). **e** Rats treated with IPC-EtOH (200 mg/kg) showed nearly normal histoarchitecture of the three layers compared to the AlCl_3_ group except for a few pyknotic glial cells (black arrow head) in the molecular layer (M)

The hippocampal tissue of control rats consisted of three normal layers: molecular, Purkinje, and polymorphic. Molecular and polymorphic layers are composed of neurons and glial cells on a neuropil background, whereas the Purkinje cell layer is formed of triangular shaped neurons with vesicular large nuclei arranged in rows (Fig. 8a). In contrast, hippocampal sections of rats treated with AlCl_3_ revealed various alterations in the histoarchitecture compared to control rats, such as pyknosis of glial cells with pericellular space in the molecular layer, disarrangement of Purkinje cells that appeared shrunken and pyknotic with perineuronal space (Fig. 8b). However, the hippocampal tissue of rats treated with donepezil hydrochloride as a reference drug revealed maintenance of nearly normal arranged Purkinje cells that appeared triangular and had vesicular nuclei compared to the AlCl_3_ group, except for a few pyknotic Purkinje cells and glial cells with pericellular spaces (Fig. 8c). Otherwise, the hippocampus of rats treated with IPC-EtOH (100 mg/kg) showed nearly normal arranged Purkinje cells but there were few degenerated Purkinje cells, and pyknotic glial cells in the molecular layer (Fig. 8d). Furthermore, the hippocampus of rats treated with a high dose of IPC-EtOH (200 mg/kg) showed nearly normal histoarchitecture of the three layers compared to the AlCl_3_ group, with the exception of a few pyknotic glial cells in the molecular layer (Fig. 8e).

### Results of immunohistochemical observations of COX-2

**Fig. 9:**
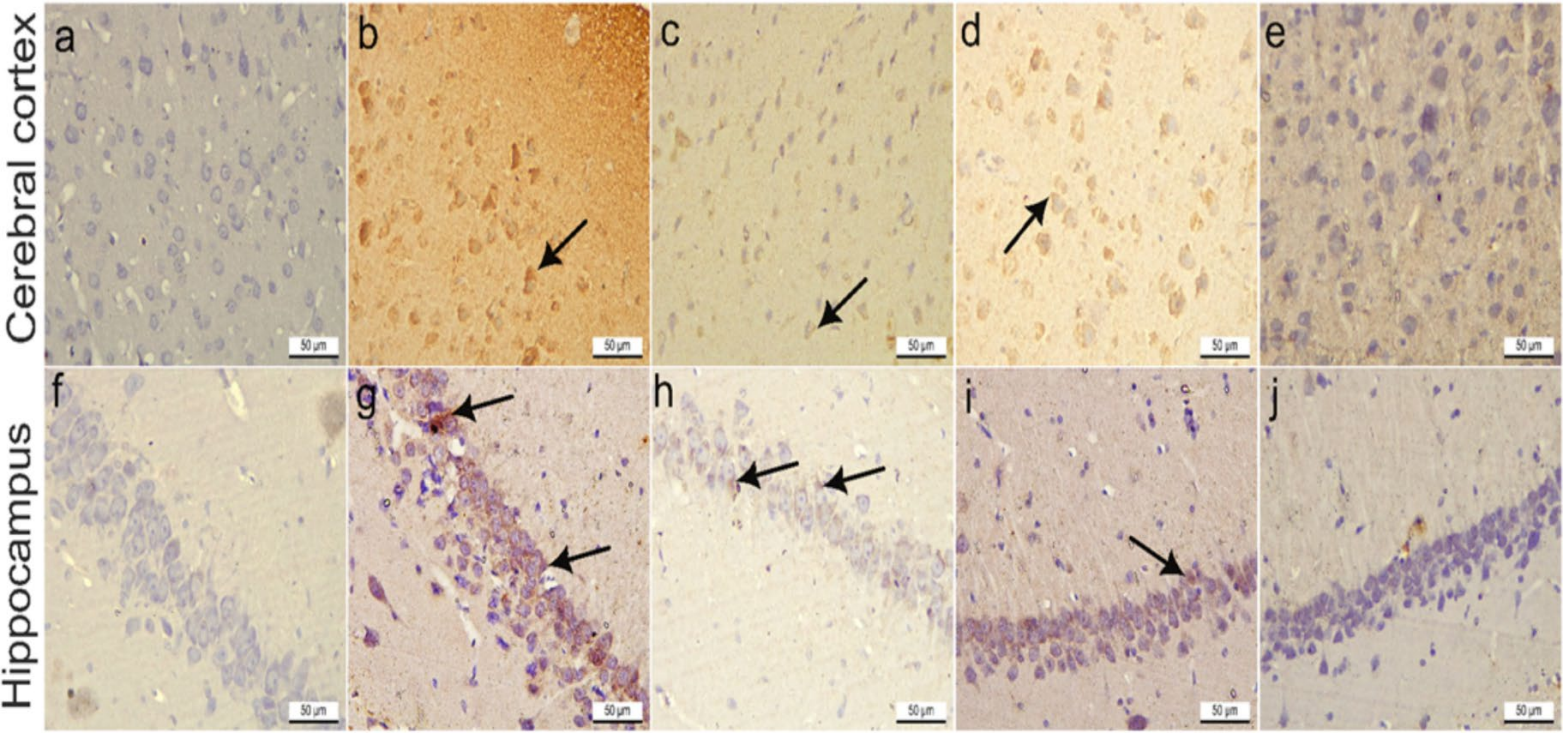
Immunohistochemistry COX-2 stained cerebral cortex sections **a**: **e** and hippocampal sections **f**: **j** from adult Wistar rats.X400. **a** Cerebral cortex sections and (**f**) hippocampal sections from control rats showed negative COX-2 immunostaining. **b** Cerebral cortex sections and **g** hippocampal sections of rats treated with AlCl_3_ revealed strong positive COX-2 immunoexpression (black arrows) compared to the control group. **c** Cerebral cortex sections and **h** hippocampal sections of rats treated with donepezil hydrochloride as a reference drug displayed mild COX-2 immunoreactivity (black arrows) compared with AlCl_3_-treated group. **d** Cerebral cortex sections and **i** hippocampal sections of rats treated with IPC-EtOH (100 mg/kg) showed moderate COX-2 immunostaining (black arrows). **e** Cerebral cortex sections and **j** hippocampal sections of rats treated with IPC-EtOH (200 mg/kg) revealed negligible COX-2 immunostaining

**Fig. 10:**
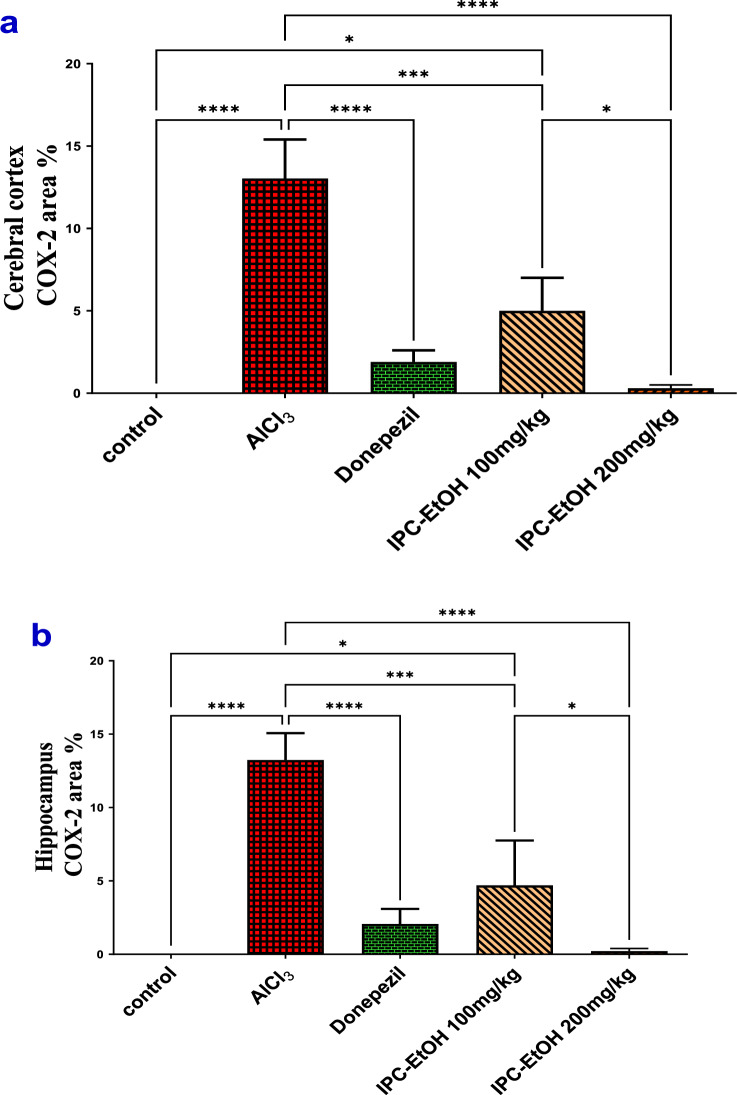
Effect of AlCl_3_, AlCl_3_ plus donepezil hydrochloride as a reference drug, AlCl_3_ plus low dose of IPC-EtOH (100 mg/kg), and AlCl_3_ plus IPC-EtOH (200 mg/kg) on the percentage area covered by cyclooxygenase-2 (COX-2)-positive immunoreactive cells within brain tissue. The results are expressed as mean ± SD. *p* value ≤ 0.0001

Immunohistochemically, COX-2 stained cerebral cortex and hippocampal sections from control rats displayed negative immunostaining (Fig. 9a and f). On the other hand, cerebral cortex and hippocampal sections of rats treated with AlCl_3_ showed strong positive COX2 immunoexpression in the neuronal cytoplasm, which was significantly increased by 13 and 13.2 respectively compared to the control group (Fig. 9b, g**,** and Fig. 9a, b). Meanwhile, the cerebral cortex and hippocampal sections of rats treated with donepezil hydrochloride as a reference drug revealed mild COX2 immunostaining that was significantly decreased by 1.9 and 2 compared to the AlCl_3_ group (Fig. 9c, h, and Fig. 10a, b). Otherwise, the cerebral cortex and hippocampal sections of rats treated with IPC-EtOH (100 mg/kg) displayed moderate COX-2 immunoreactivity, which was significantly reduced by 5 and 4.7 compared to the AlCl_3_ group (Fig. 9d, I and Fig. 10a, b). The cerebral cortex and hippocampal sections of rats treated with a high dose of IPC-EtOH (200 mg/kg) showed negligible COX-2 immunostaining in the neuronal cytoplasm, which decreased significantly by 0.3, 0.2 compared to the AlCl_3_ group (Fig. 9e, j and Fig. 10a, b).

## Discussion

Neurodegeneration is a feature of AD, it is related to aging and is mostly a manifestation of cerebrovascular diseases that may occur because of oxidative stress and neuro-inflammation, leading to neurodegeneration and cognitive dysfunction (Abou Baker et al. 2020). In the study of Sharma et al. (Sharma et al. 2007), it was reported that the 172 mg/kg/day of AlCl_3_ given orally to rats for 10 weeks leads to the occurrence of brain oxidative stress (Sharma et al. 2012). In the present study, an animal model with symptoms mimicking AD in humans was induced by 172 mg/kg/day of AlCl_3_, which were manifested by significant reduction of rat movements across the open field in comparison to normal control rats, which was the same case in the study of Abou Baker et al. (Abou Baker et al. 2020), in addition to the absence of rat alternation in the T-maze test in our study. This denotes marked deterioration in the psychological state and cognitive abilities of rats, which may be due to deficiency of acetylcholine (Ach) in brain tissue, in addition to accumulation of β-amyloid proteins and the development of neurofibrillary tangles, together with neuronal degeneration, which are the most important elements in AD. For this reason, the main goal of treating Alzheimer's disease is acetylcholinesterase inhibitors such as donepezil, rivastigmine, and galantamine (Karam et al. 2014).

In the present study, donepezil-treated groups showed a significant increase in rats’ movement in the open field and alternations in the T-maze tests, together with improvement in all biochemical parameters, as it caused a significant reduction in β-amyloid, tau protein levels, β-secretase levels, and GS3kb levels, and a significant increase in SOD and CAT levels when compared to the positive control group. These results are consistent with the previous results of Elkomy et al. (Elkomy et al. 2021), who reported that donepezil treatment of rats protected from AD development because of the administration of AlCl_3_, improved symptoms of AD significantly and significantly elevated the levels of GSH and SOD, indicating that its antioxidant capacity contributed to its ability to improve the prognosis of AD.

Therefore, treatment of rats in our study with natural products that possess antioxidant and subsequently anti-inflammatory activities was considered in our study. Fortunately, the IPC-EtOH within the used doses at 100 and 200 mg/kg showed promising effects on Ach levels and exhibited a significant reduction in β-amyloid protein levels, tau protein levels, β-secretase, and GS3kb, as well as a significant increase in SOD and GSSG compared to the positive control group, together with a significant reduction in neurofibrillary tangles and neuronal degeneration. The neuroprotective effects of IPC-EtOH are attributed to its antioxidant and anti-inflammatory activities, which retard cerebrovascular aging and the emergence of AD as confirmed by immunohistochemical examination that revealed that AD rats treated with IPC-EtOH showed a significant reduction in the inflammatory marker COX-2 immunoexpression, which confirms the IPC-EtOH effective role as an anti-inflammatory agent.

Tau is a microtubule-associated protein that plays a crucial role in the stability and function of neuronal microtubules. hyperphosphorylated tau as in Alzheimer’s disease, leading to the formation of neurofibrillary tangles, which are a hallmark pathological feature of the disease. The hyperphosphorylation and accumulation of tau disrupts the normal function of microtubules, leading to the collapse of the neuronal cytoskeleton and contributing to neuronal dysfunction and degeneration that is manifested by cognitive deficits and tightly related to mitochondrial dysfunction and neuroinflammation via multiple pathways (Ye et al. 2024).

AlCl_3_ exposure has been shown to increase the activity of glycogen synthase kinase-3β (GSK3β), which is a key kinase responsible for the hyperphosphorylation of tau (Sanajou et al. 2023). In the present study, the i.p. administration of AlCl_3_ exerts the hyperphosphorylation of tau and elevated β-secretase levels, which is known as BACE1 (Beta-Site Amyloid Precursor Protein Cleaving Enzyme 1), an enzyme responsible for the initial cleavage of the amyloid precursor protein (APP), leading to the generation of amyloid-beta (Aβ) peptides. Recent research affirmed the presence of a corresponding relation between the activity of BACE1 and the production of Aβ, the latter represents a central pathological hallmark of Alzheimer’s disease (Bazzari and Bazzari 2023). Recent studies have shown that AlCl_3_ exposure can upregulate the expression and activity of BACE1 in the brain, contributing to the increased production of Aβ peptides. Its accumulation leads to the formation of amyloid plaques, which are another characteristic feature of Alzheimer’s disease pathology (Abu-Elfotuh et al. 2024; Elreedy et al. 2023; Hampel and Shen 2009).

Oxidative stress plays an integral role in the deterioration of the physiological status of most tissues, especially neural cells. The increased oxidative stress can trigger signaling pathways that contribute to the development of AD pathology, including the accumulation of amyloid-beta (Aβ) and the hyperphosphorylation of tau protein (Cai et al. 2011b). Mitochondrial dysfunction occurred during the oxidative stress that could be attributed to the depletion of SOD, catalase, the imbalance of the GSH/GSSG ratio, etc. (Santos et al. 2007). SOD is an important antioxidant enzyme that plays a crucial role in the first line of defense against ROS by dismutating superoxide radicals (O₂⁻) to hydrogen peroxide (H₂O₂) and oxygen (O₂), which can otherwise react with and damage cellular components (Pandhair and Sekhon 2006). The role of SOD is supported by catalase, where the latter could catalyze the decomposition of hydrogen peroxide (H₂O₂) into water (H₂O) and oxygen (O₂) (Milgrom 2016). The oxidation of GSH to GSSG in the brain represents the obvious mark of oxidative stress that led to neurodegeneration (El-Hawary et al. 2021). In the existing work, AlCl_3_ treatment resulted in the elevation of GSSG with the depletion of SOD and catalase, as previously stated by Ngoumen et al. and Rashwan et al. (Ngoumen et al. 2023; Rashwan et al. 2018). Donepezil has antioxidant activity that ameliorated the oxidative stress manifested by elevation of CAT and SOD and decreasing the GSSG levels (Uddin et al. 2016). The tight association between the oxidative stress and apoptosis could be translated by the following image: the oxidative stress could provoke the apoptotic pathways (Chen et al. 2008). FOXO3a is a transcription factor that plays a crucial role in regulating oxidative stress and apoptosis. In Alzheimer’s disease, oxidative stress is a significant factor contributing to neuronal damage (Zhao et al. 2020). FOXO3a can be phosphorylated by GSK3β, which affects its activity and localization within the cell and is involved in various cellular processes, including the regulation of tau phosphorylation (Liu et al., 2024). Although donepezil has a distinct mechanism as a choline esterase inhibitor, it could actively regulate FOXO3a expression (Zhao et al. 2020). The effect of donepezil on GSK3β is indirect, where donepezil inhibits cholinesterase, that leads to increased acetylcholine levels that trigger the activation of muscarinic acetylcholine receptors which, in turn, inhibit the activity of GSK3β and control phosphorylation of tau protein (Sayas and Ávila 2021).

In the current study, AlCl_3_ treatment resulted in various histopathological alterations in the brain tissue histoarchitecture of adult Wistar rats, such as shrinkage and pyknosis of pyramidal space in the hippocampal tissue, compared to control rats and these findings were consistent with those reported previously in both the cerebral cortex and in the hippocampus (Elgendy et al. 2008; Liaquat et al. 2019; Shehata et al. 2023; Taiye et al.; Adelodun et al. 2021; Kamel and Mostafa 2013). Irnidayanti and Aprilyanti (2021) reported that neurodegenerative changes induced by aluminum toxicity may be attributed to oxidative stress damage. Moreover, brain tissue is susceptible to reactive oxygen species (ROS) and lipid peroxidation (Azib et al. 2019; Irnidayanti and Aprilyanti 2021) owing to its high consumption of oxygen, which in turn becomes a factory for reactive oxygen species (ROS) as well as the occurrence of neurodegeneration (Singh et al. 2019). On the other hand, treatment with donepezil hydrochloride as a reference drug revealed marked maintenance of the nearly normal structure of most neurons in both the cerebral cortex and hippocampus tissues compared to aluminum chloride-treated rats. These findings agree with those of Shehata et al. (2023)**,** who stated that the cortical and hippocampal histopathological parameters were improved by the treatment of rats with donepezil, owing to its anti-inflammatory and antioxidant neuroprotective effects (Shehata et al. 2023).

Meanwhile, treatment of aluminum chloride-treated rats with IPC-EtOH resulted in marked preservation of the nearly normal histoarchitecture of the cerebral cortex and hippocampal tissues, which was enhanced by increasing the dose of IPC-EtOH to 200 mg/kg. This neuroprotective effect may be attributed to the antioxidant effect of IPC-EtOH due to the presence of antioxidants like polyphenols and flavonoids in its.

leaves, stems, and flowers (Khatiwora et al. 2010).

In this study, rats treated with aluminum chloride showed neuroinflammation, as evidenced by the intense immunohistochemical expression of cyclooxygenase 2 (COX-2) in the cerebral cortex and hippocampus compared to control rats. These results were confirmed by the results of Maksoud et al. (2020)**,** who reported elevation of IL-2, IL-6, and serum tumor necrosis factor (TNFα) concentration in the AlCl_3_ group. Moreover, Elhadidy et al. (2018) reported elevated levels of cortical and hippocampal TNF-α in AlCl_3_-treated rats. However, rats treated with donepezil hydrochloride showed a significant decrease in COX-2 immunostaining compared to the AlCl_3_ group. Kim et al. (2021) reported that donepezil had anti-inflammatory activity by inhibiting microglia activation, ROS, and pro-inflammatory cytokine production. In addition, treatment of aluminum chloride-treated rats with IPC-EtOH led to a significant decrease in COX-2 immunoexpression, which was increased by the high dose (200 mg) compared to that in the AlCl_3_ group (Elhadidy et al. 2018; Maksoud et al. 2020; Kim et al. 2021).

The present neuroprotective effects of IPC-EtOH might be attributed to its significant anti-inflammatory capabilities, which are supplied via its metabolites (Ruchi et al. 2009; Khalid et al. 2011). IPC-EtOH’s current chemical profile showed the existence of many different bioactive metabolites, specifically flavonoids, pyrrolizidine alkaloids, and other polyphenolic components. Several natural pyrrolizidine alkaloids isolated from plants were documented to have essential activity as AChE inhibitors (Wei et al. 2021). In AD, acetylcholinesterase (AChE) is associated with neurofibrillary tangles and a buildup of external Aβ deposition. Provided medications for AD work primarily by blocking AChE to lessen effects or slow the illness’s progression (Aryal et al. 2022).

Alkaloids may have neuroprotective effects by reducing a number of cellular processes, including the functioning of the AChE enzyme, raising the concentration of GABA, a neurotransmitter that is inhibitory in the mammalian brain, by partially inhibiting NMDA receptors in the brain, encouraging cellular autophagy, and many other processes (Hussain et al. 2018). Also, the pyrrolizidine alkaloids may provide this significant anti-inflammatory impact. They have been shown to reduce LPS-induced COX-2 expression in macrophages by suppressing NF-kB expression (Chauhan et al. 2022).

Furthermore, a variety of mechanisms were proposed to explain how polyphenolic compounds and flavonoids can reduce the buildup of Aβ (Minocha et al. 2022). Flavonoids have the ability to directly prevent aggregation (Hole and Williams 2020), inhibit GSK-3β-mediated tau phosphorylation (Pierzynowska et al. 2019), and decrease Aβ formation (Uddin et al. 2020; Ibrahim et al. 2024). Multiple investigations have demonstrated that flavonoids and their metabolites can interact with lipid kinase-signaling cascades and protein kinase and exert beneficial impacts on neurological processes (Wang et al. 2008). These neuronal signaling pathways include the NF-κB-light-chain enhancement of the activated B cell (Singh et al. 2010), the mitogen activated kinase (MAPK) signaling pathways (Uddin et al. 2020; Ibrahim et al. 2024), phosphoinositide-3-kinase (PI3K)/Akt (Singh et al. 2010), protein kinase C (PKC), and tyrosine kinase (Obulesu and Rao 2011). Furthermore, via interactions with membrane receptors and MAPK kinases including MAP kinase 1 (MEK1) and MEK2, flavonoids and their metabolites might effectively engage through MAPK signaling mechanisms (Ibrahim et al. 2024; Wang et al. 2008).

The suppression of the hyperphosphorylation and acetylation of tau was described as a main mechanism of decreasing the head injury and dementia (Amato et al. 2019; Zhao and Zlokovic 2021; Pluta et al. 2022; Sharifi-Rad et al. 2022). Numerous polyphenolic metabolites originating from plants, including ferulic acid, caffeoylquinic acid, caffeic acid, chlorogenic acid, and hydroxycinnamic acids, have been shown to possess substantial capacities to inhibit tau hyperphosphorylation and acetylation in both in vitro and in vivo models (Wesseling et al. 2020; Zhao and Zlokovic 2021; Angeloni et al. 2021; Sharifi-Rad et al. 2022). Several bioactive derivatives of these compounds, including feruloyl caffeoyl quinic acid, 3-*P*-coumaroyl-4-caffeoylquinic acid, tricaffeoyl quinic acid, diferuloyl quinic acid, 5-feruloylquinic acid, quinic acid, 3-CQA (neochlorogenic acid), 5-CQA (chlorogenic acid), and 4-CQA (cryptochlorogenic acid), were found in the current IPC-EtOH profiling. Each of the above components significantly inhibited the hyperphosphorylation and acetylation of tau by (i) binding to tau protein and thus inhibits its ability to form tau oligomers, (ii) potentially decreasing tau-induced cytotoxicity, (iii) modulating of the tau hyperphosphorylation through inhibition of kinases like GSK-3β (Sul et al. 2009; Singh et al. 2021b), (iv) preventing tau aggregation into insoluble tangles, and (v) modulatines oxidative stress, which is a known contributor to tau pathology (Singh et al. 2021b).

Flavonoids have been shown to affect tau aggregation and phosphorylation in multiple ways including (i) reduction of the activity of tau-associated kinases like GSK-3β, (ii) promotion of the tau’s dephosphorylation through activation of protein phosphatases (Singh et al. 2021b; Baptista et al. 2014), (iii) modulating tau conformational changes (Monteiro et al. 2018), and (iv) modulation of cellular signaling pathways, including the AMPK and SIRT1 pathways, which are involved in regulating tau pathology (Chua et al. 2017).

Several studies suggest that alkaloids may modulate tau aggregation and phosphorylation. For instance, quinoline and isoquinoline alkaloids have been shown to interfere with tau fibrillogenesis in vitro. Quinoline derivatives can disrupt the aggregation of tau by binding to tau’s microtubule-binding domains, preventing the formation of toxic tau oligomers (Li et al. 2023). In addition, these components have been demonstrated to inhibit tau hyperphosphorylation by interacting with kinases such as glycogen synthase kinase 3 beta (GSK-3β), a key enzyme involved in tau phosphorylation in addition to suppressing tau aggregation by binding to tau fibrils and preventing further polymerization (Navarrete et al. 2012).

The AD pathways-cholinergic deficiency, oxidative stress, and inflammation-have been shown to be significantly associated with the factors that trigger the processing of amyloid precursor proteins, particularly tau protein acetylation, hyperphosphorylation and aggregation, along with the β and γ secretases (Ahmed et al. 2021; Tuzimski and Petruczynik 2022). Thus, IPC-EtOH's capacity to control inflammation and oxidative stress was enhanced by the activity of the annotated alkaloids, flavonoids, and polyphenolic components, which further reduced tau acetylation, hyperphosphorylation, and aggregation.

## Conclusion

In summary, Alzheimer’s disease neuropathogenesis, the main risk factors of the disease and the role of naturally occurring compounds and their synthetic derivatives in AD management. The current study implies that IPC-EtOH may be a viable treatment for reducing aluminum-induced neurotoxicity, but more research is required to determine its exact modes of action. These results highlight the potential of *I. carnea* ethanol extract as a promising treatment for AD by showcasing its neuroprotective and memory-enhancing qualities in rats with memory impairment caused by AlCl_3_.

## Supplementary Information

Below is the link to the electronic supplementary material.

Supplementary file1 (DOCX 203 KB)

## Data Availability

The datasets generated during and/or analyzed during the current study are available from the corresponding author on reasonable request.
