# Supplementary material for: Unveiling the neuroprotective potential of Ipomoea carnea ethanol extract via the modulation of tau and β-secretase pathways in AlCl3-induced memory impairment in rats in relation to its phytochemical profiling
Source: Inflammopharmacology. 2025 Mar 12;33(4):2043–68. doi: 10.1007/s10787-025-01687-0 (PMC11991951; doi:10.1007/s10787-025-01687-0)
Supplement: Supplementary file 1 — Supplementary file1 (DOCX 203 KB) [file 10787_2025_1687_MOESM1_ESM.docx]

**Supporting data**

**A**

**B**

**Figure 1S**: Representative UPLC-ESI-MS of *Ipomoea carnea* ethanol extract (IPC-EtOH) including positive mode (A) and negative mode (B).

**Figure 2S**. Structures of Pyrrolizidine alkaloids (PAs) and their different forms, R1 and R2 represent necic acids.

**Figure 3S**. Pyrrolizidine alkaloids (PAs) groups according to necine bases.

|  | | |  | | | |  | | | |
| --- | --- | --- | --- | --- | --- | --- | --- | --- | --- | --- |
| **Compd** | **R_1_** | **R_2_** | **Compd** | **R_1_** | | **R_2_** | **Compd** | **R_1_** | | **R_2_** |
|  |  |  | **4** | H | | H |  |  | |  |
| **1** | H | H | **11** | CH_3_ | | H | **5** | H | | H |
| **7** | CH_3_ | H | **14** | H | | COCH_3_ | **12** | CH_3_ | | H |
| **16** | H | COCH_3_ | **17** | CH_3_ | | COCH_3_ |  |  | |  |
|  | | |  | | | |  | | | |
| **Compd** | **R_1_** | **R_2_** | **Compd** | | **R_1_** | **R_2_** | **Compd** | | **R_1_** | **R_2_** |
| **6** | H | H | **3** | | H | H |  | |  |  |
| **13** | CH_3_ | H | **8** | | CH_3_ | H | **2** | | H | H |
| **15** | H | COCH_3_ | **10** | | H | COCH_3_ | **9** | | CH_3_ | H |
| **18** | CH_3_ | COCH_3_ | **19** | | CH_3_ | COCH_3_ |  | |  |  |
|  | | |  | | | |  | | | |
| **66** | | | **72** | | | | **73** | | | |
|  | | | | | | |  | | | |
| **74** | | | | | | | **78** | | | |

**Figure 4S.** Structures of some identified compounds.

**Figure 5S**. MS/MS fragments of different Pyrrolizidine alkaloids (PAs), distinctive product ions can be produced via MS/MS.
